# Fast‐Charging Solid‐State Li Batteries: Materials, Strategies, and Prospects

**DOI:** 10.1002/adma.202417796

**Published:** 2024-12-25

**Authors:** Jing Yu, Yuhao Wang, Longyun Shen, Jiapeng Liu, Zilong Wang, Shengjun Xu, Ho Mei Law, Francesco Ciucci

**Affiliations:** ^1^ College of Chemistry and Chemical Engineering Zhongkai University of Agriculture and Engineering Guangzhou 510225 China; ^2^ Department of Mechanical and Aerospace Engineering The Hong Kong University of Science and Technology Hong Kong 999077 China; ^3^ School of Advanced Energy Sun Yat‐Sen University Shenzhen 518107 China; ^4^ Chair of Electrode Design for Electrochemical Energy Systems University of Bayreuth 95448 Bayreuth Bavaria Germany; ^5^ Bavarian Center for Battery Technology (BayBatt) 95447 Bayreuth Bavaria Germany

**Keywords:** fast charging, interface/interphase chemistry, Li^+^ transport, solid‐state batteries

## Abstract

The ability to rapidly charge batteries is crucial for widespread electrification across a number of key sectors, including transportation, grid storage, and portable electronics. Nevertheless, conventional Li‐ion batteries with organic liquid electrolytes face significant technical challenges in achieving rapid charging rates without sacrificing electrochemical efficiency and safety. Solid‐state batteries (SSBs) offer intrinsic stability and safety over their liquid counterparts, which can potentially bring exciting opportunities for fast charging applications. Yet realizing fast‐charging SSBs remains challenging due to several fundamental obstacles, including slow Li^+^ transport within solid electrolytes, sluggish kinetics with the electrodes, poor electrode/electrolyte interfacial contact, as well as the growth of Li dendrites. This article examines fast‐charging SSB challenges through a comprehensive review of materials and strategies for solid electrolytes (ceramics, polymers, and composites), electrodes, and their composites. In particular, methods to enhance ion transport through crystal structure engineering, compositional control, and microstructure optimization are analyzed. The review also addresses interface/interphase chemistry and Li^+^ transport mechanisms, providing insights to guide material design and interface optimization for next‐generation fast‐charging SSBs.

## Graphical abstract

This review addresses challenges and recent advances in fast‐charging solid‐state batteries, focusing on solid electrolyte and electrode materials, as well as interfacial chemistries. The role of multiscale modeling and simulation in understanding Li^+^ transport and interfacial phenomena is emphasized, providing insights into materials, strategies, and future prospects for high‐performance, fast‐charging solid‐state batteries.

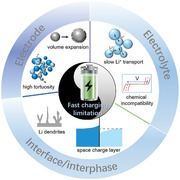

## 1 Introduction

Since their commercial introduction in 1991, rechargeable Li‐ion batteries (LIBs) have become the dominant power source for portable electronics, electric vehicles (EVs), and drones. However, the current generation of LIBs has struggled to meet increasing market demands due to energy density limitations, safety concerns, and, importantly, rate capability constraints.^[^ ^1^ ^]^ High‐rate operation has been found to hasten battery degradation, causing a capability decline due to the slow Li^+^ diffusion in the electrodes and electrolyte, along with sluggish intercalation kinetics. Additionally, heat generated during fast charging/discharging presents challenges in dissipating heat uniformly, leading to accelerated degradation and safety concerns. In fact, an EV's driving discharge rate typically occurs within 2–5 h to maximize performance, longevity, and safety, while recharging EV batteries significantly surpasses the time required to refuel conventional, fossil fuel‐powered vehicles. Consequently, fast charging has become a pivotal factor in accelerating EV market adoption and, by extension, has driven advancements in battery technology.

**Figure 1:**
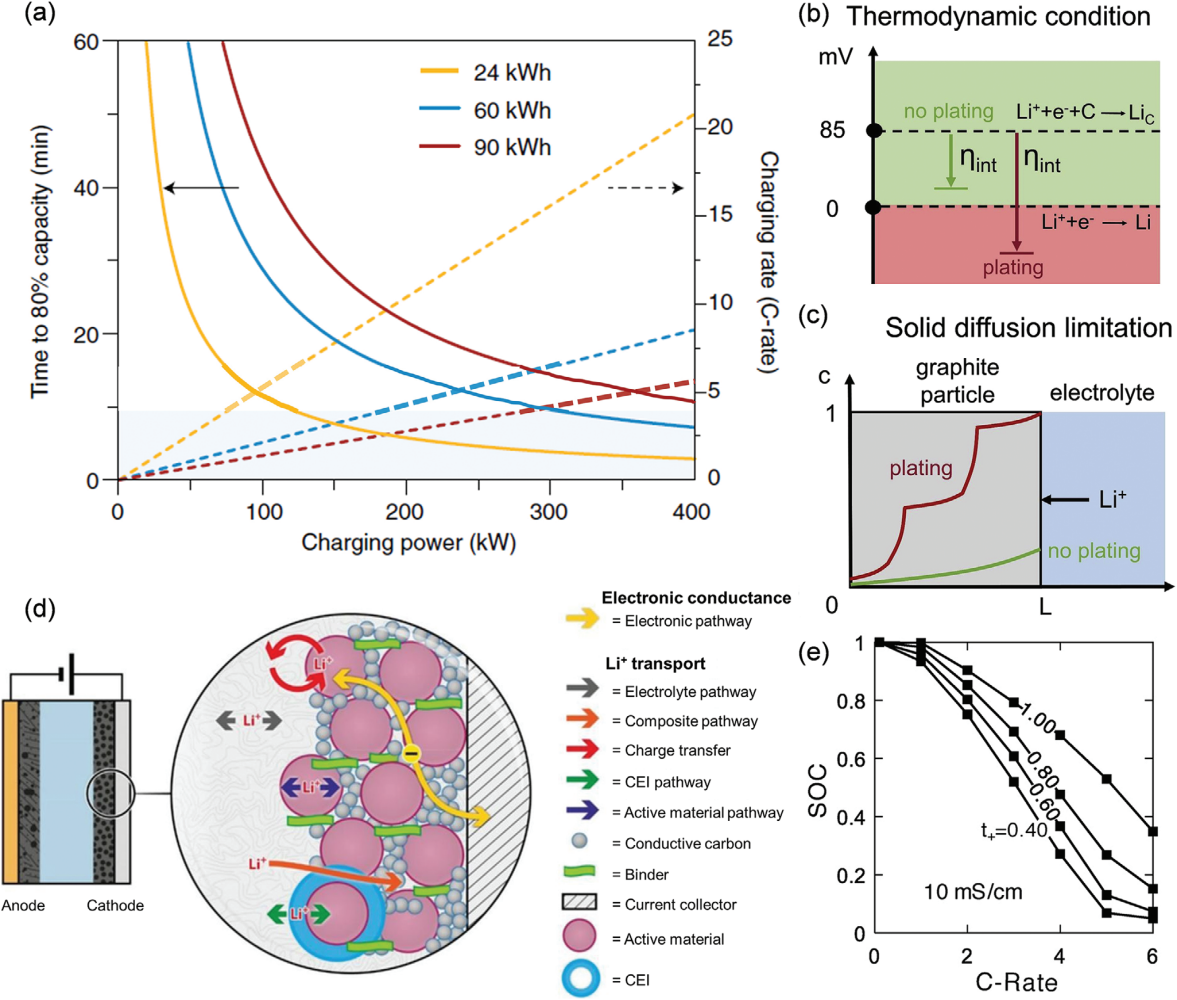
a) Relationship between recharge time to 80% state of charge (solid lines), corresponding charging rate (C‐rate, dashed lines), and charging power for three different battery pack sizes (24, 60, and 90 kWh). The shaded region represents charging powers that meet the US Advanced Battery Consortium's goals for fast‐charge EV batteries. Reproduced with permission.^[^ ^3^ ^]^ Copyright 2019, Springer. b) Thermodynamic conditions for Li plating on the anode (vs Li/Li^+^) under varying current and Li^+^ insertion kinetics. The green arrow indicates small current favoring intercalation, while the red arrow indicates large current conditions favoring Li plating. c) Schematic illustration of how sluggish Li^+^ diffusion in the solid electrode leads to Li concentration saturation at the electrode surface, promoting Li plating. Panels b,c) Reproduced with permission.^[^ ^4^ ^]^ Copyright 2021, Elsevier. d) Schematic representation of an LIB cathode including the kinetic processes and Li^+^ pathways. Reproduced with permission.^[^ ^5^ ^]^ Copyright 2016, The Electrochemical Society. e) Attainable SOC as a function of C‐rate for an electrolyte with *σ* = 10 mS cm^−1^ and varying $t_{Li^{+}}$. Panels d,e) Reproduced with permission.^[^ ^7^ ^]^ Copyright 2017, American Chemical Society.

In 2023, the US Advanced Battery Consortium established a target of reaching 80% state of charge (SOC) in 15 min for fast‐charge EV batteries, regardless of pack size.^[^ ^2^ ^]^ **Figure** **1**a presents a theoretical plot demonstrating the relationship between recharge time to 80% SOC, charging rate, and charging power for three different battery pack sizes.^[^ ^3^ ^]^ For a fixed pack size, charging rate increases, or charging time decreases with higher charging power. The shaded area in Figure 1a indicates charging powers that align with the US Advanced Battery Consortium's goals for fast‐charge EV batteries. Achieving a 15‐min recharge for larger packs (e.g., 90 kWh) necessitates a charging power of ≈300 kW, while smaller packs (e.g., 24 kWh) can meet the fast‐charging target at ≈80 kW. Correspondingly, a charging rate of 4C or higher, is equal to a nominal charge time of 15 min or less.

The current generation of LIBs cannot normally be operated under a high charging rate. Taking commonly adopted graphite in commercial LIBs as an example, under slow charging rates, Li^+^ has sufficient time to intercalate deeply into the anode's active material. However, at high charging rates, Li^+^ intercalation becomes a bottleneck, limiting active material utilization, while Li plating reaction becomes thermodynamically possible (Figure 1b).^[^ ^4^ ^]^ Consequently, charging at excessively high rates and repeated Li plating concurrent with intercalation can potentially accelerate the growth of Li dendrite (Figure 1c).^[^ ^4^ ^]^ The cathode typically comprises nonactive components, including a binder for boosting mechanical robustness and structural integrity, conductive carbon for improving electronic conductivity, and an Al current collector that serves as a substrate for the electrode coating. The intricate nature of a composite electrode presents a significant challenge due to the diverse kinetic processes (Figure 1d), which influence the cathode's internal resistance.^[^ ^5^ ^]^ Furthermore, Li^+^ diffusion is influenced by the concentration gradient between the electrode and the liquid electrolyte. Liquid electrolytes, while offering high ionic conductivity (σ) and good interfacial contact with electrodes, typically exhibit low Li^+^ transference numbers $\left(t_{Li^{+}}\right)$, often ranging from 0.2 to 0.4.^[^ ^6^ ^]^ This low $t_{Li^{+}}$ inevitably results in Li^+^ accumulation and depletion at the electrodes, leading to concentration gradients during cycling.^[^ ^7^ ^]^ Figure 1e illustrates the influence of $t_{Li^{+}}$ on SOC for an electrolyte with σ = 10 mS cm^−1^, highlighting the advantage of high $t_{Li^{+}}\;$ at high C‐rates.^[^ ^7^ ^]^ Additionally, charging can elevate battery temperatures, leading to parasitic reactions that may cause thermal runaway and even catastrophic failure.^[^ ^3^ ^]^ These phenomena are exacerbated under fast‐charging conditions, contributing to possible dendrite growth and side reactions, increased interfacial resistances, and decreased battery capacity. Extensive research has focused on achieving fast charging by leveraging thermal management,^[^ ^8^ ^]^ optimizing charging protocols,^[^ ^9^ ^]^ and introducing innovative materials and structure design.^[^ ^10^ ^]^ However, current LIBs technology, which still relies on organic liquid electrolytes, faces significant challenges in realizing fast charging without compromising safety and performance.

A promising pathway to address the challenges hindering widespread fast‐charging adoption lies in the development of solid‐state batteries (SSBs). By replacing flammable organic liquid electrolytes with nonflammable solid electrolytes (SEs), SSBs offer enhanced safety, a critical factor in fast‐charging applications. Additionally, the generally higher thermal stability of SEs compared to liquid electrolytes allows them to withstand the elevated temperatures achieved during fast charging.^[^ ^11^ ^]^ In contrast, batteries with liquid electrolytes experience accelerated degradation above 60 °C, limiting their capabilities during fast charging.^[^ ^9a^ ^]^ SEs are a promising alternative for enabling the use of Li metal batteries. The high theoretical specific capacity (3860 mAh g⁻¹) and low electrochemical potential (−3.04 V vs the standard hydrogen electrode) of Li metal allow SSBs to achieve higher energy densities. Utilizing a higher‐capacity anode reduces the mass loading of active materials, and thus the charge carrier transport distance, which is crucial for fast charging. Furthermore, SEs’ higher $t_{Li^{+}}$ (e.g., close to 1 for ceramic SEs) enables predominant Li^+^ transport, effectively minimizing concentration gradients during charge and discharge cycles when compared to liquid electrolytes (e.g., $t_{Li^{+}}$ = ≈0.2–0.4).^[^ ^7^ ^]^ Moreover, the mechanical rigidity of certain SEs, such as inorganic ceramics, can delay Li dendrite growth, enhancing stability during fast charging.^[^ ^12^ ^]^

**Figure 2:**
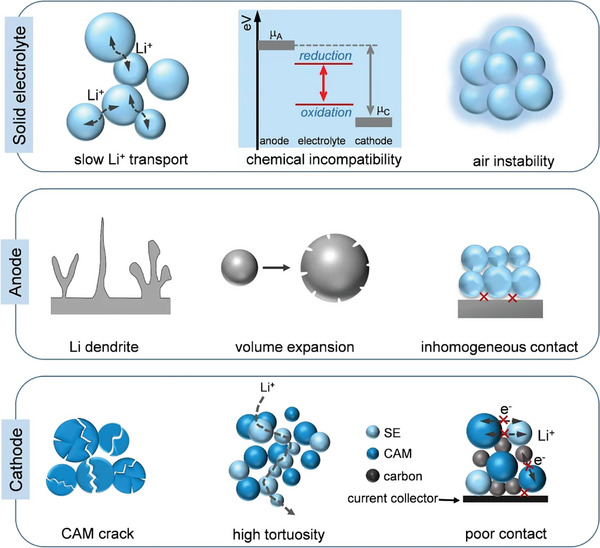
Challenges of the main components (SE, anode, and cathode) for fast‐charging SSBs.

However, fast‐charging SSBs have not been commercialized because of their sluggish ion transport rate within solids and the poor interfacial compatibility and adhesion resulting from the rigidity of SEs. To this end, this article first summarizes the challenges related to key components of SSBs during fast charging (**Figure** **2**), and provides a comprehensive overview of recent advancements in electrolyte materials, focusing on inorganic ceramic electrolytes (ICEs), solid polymer electrolytes (SPEs), and inorganic‐polymer composite electrolytes (IPCs). Meanwhile, the review examines electrode active materials and interfacial chemistries tailored to enhance ion and electron transport kinetics within electrodes and facilitate efficient charge transfer across interfaces in fast‐charging SSBs. Furthermore, the review discusses the substantial insights derived from computational methodologies, including density functional theory (DFT), molecular dynamics (MD) simulations, high‐throughput screening (HTS), continuum models, and machine learning (ML) techniques. These computational approaches not only enable the rational design of materials but also elucidate the fundamental mechanisms governing fast‐charging SSBs. The analysis provided herein underscores the critical role of materials, interfacial chemistries, and computational methods in developing high‐performance fast‐charging SSBs. It is anticipated that the knowledge gained from this review will help direct future research endeavors toward the rational design and optimization of SSBs for fast‐charging applications.

## 2 Challenges for Fast‐Charging Solid‐State Batteries

Several key challenges (Figure 2) must be overcome to unlock the full potential and enable widespread adoption of fast‐charging SSBs. These challenges primarily originate from i) the limited Li^+^ diffusion through the SE, electrode, and interface, ii) the structural instability of the SE and cathode, iii) the Li dendrite growth and safety concerns under large current density, and iv) poor physical contact and large interfacial resistance.

### 2.1 Low Ionic Conductivity and Instability of Solid Electrolytes

Most SEs exhibit insufficient ionic conductivity, which is a critical barrier to achieving fast‐charging SSBs. The ionic conductivity of SEs depends heavily on diffusion pathways, as determined by inter‐site hopping in ICEs or segmental motion of polymer chains in PEs. While ICEs often demonstrate high bulk conductivity, their high grain boundary resistance significantly reduces overall ionic conductivity, impeding Li^+^ transport.^[^ ^13^ ^]^ Most SPEs are crystalline at room temperature (RT), making Li^+^ migration through their polymer crystals difficult, thus resulting in low ionic conductivity.^[^ ^14^ ^]^ Optimizing the design of SEs to enhance ionic conductivity is essential for achieving rapid charging speeds in SSBs, and will be discussed later. While certain ICEs, particularly sulfide, show superionic conductivity (>5 mS cm^−1^), and meet the fast‐charging requirements,^[^ ^15^ ^]^ they still face stability challenges. The electrochemical decomposition can trigger volume fluctuations and side reactions as the cell potential exceeds its stability windows.^[^ ^16^ ^]^ Moreover, the intrinsic air sensitivity characterizing most SEs including halides, sulfides, and some oxides, significantly impedes widespread SSB commercialization.^[^ ^16^, ^17^ ^]^

### 2.2 Limited Critical Current Density

There are three major challenges on the anode side, including Li dendrite growth, insufficient solid–solid contact between the anode and SE, and inhomogeneous Li^+^ deposition (Figure 2). These factors have a significant influence on critical current density (CCD), a crucial parameter for the utilization of SSBs, aiding in identifying the rate‐determining stages of Li^+^ kinetics between the Li metal and SE interfaces. Achieving a perfect interfacial contact between SE and electrode is difficult to realize. The formation and elimination of voids results in either a point‐to‐point contact or uneven face‐to‐face interfacial contact between SE and electrode, generating high local current densities that accelerate Li⁺ dendrite growth and reduce CCD. Furthermore, nonuniform anode expansion induces substantial local and global stresses, fostering uncontrolled morphological alterations, particularly when paired with rigid SEs.

Recent research has explored multiple strategies to enhance CCD performance, including interfacial resistance reduction,^[^ ^18^ ^]^ temperature optimization,^[^ ^19^ ^]^ pressure manipulation,^[^ ^20^ ^]^ and discharge parameters refinement.^[^ ^19^, ^21^ ^]^ Temperature elevation improves diffusion in both electrode and electrolyte, mitigating diffusion mismatches and improving CCD.^[^ ^22^ ^]^ For instance, at 195 °C, Li_7_La_3_Zr_2_O_12_ (LLZO) ceramic‐based Li battery failed at 530 mA cm^−2^, 1000 times higher than at RT.^[^ ^19a^ ^]^ However, elevated temperatures pose additional safety risks and may be impractical for commercial applications. Pressure‐lacking SSBs suffer from poor contact and low‐density structure, reducing volumetric energy density and creating space for parasitic reactions.^[^ ^20b^ ^]^ While appropriate pressure can improve interfacial contacts by transforming point‐to‐point contacts, excessive pressure may induce SE particle cracking and Li creep, potentially causing cell shorting.^[^ ^20^, ^23^ ^]^ Alternatively, Wen's group introduced the concept of critical areal capacity.^[^ ^19b^ ^]^

### 2.3 Poor Ion and Electron Transport of Cathodes

Unlike commercial LIBs, where liquid electrolytes readily penetrate porous electrodes, SSBs face limited contact between SE and electrode, resulting in high interfacial resistance and sluggish Li^+^ transport kinetics, thus reducing rate performance.^[^ ^24^ ^]^ The Li^+^ concentration in the space charge region created at the cathode‐SE interface is usually low, further impeding ion transport and increasing interfacial resistance.^[^ ^25^ ^]^

Within the composite cathode, transition metal ions from the active material are reduced and oxidized during battery cycling, which may induce volume changes. Even small volume changes can lead to considerable strain and local stress at the interphase between the active material and SE particles.^[^ ^26^ ^]^ Although the usage of polymer binders or optimizing the mixed SE particle size distribution may mitigate the volume changes and create a beneficial ion transport, the intricate microstructure of the composite cathode may increase tortuosity and lengthen the distance for ion and electron transport. Additionally, particle cracking due to the Li^+^ extraction and integration during cycling further reduces physical contact between the particles in the cathode, impeding ion and electron kinetics.

## 3 Solid Electrolytes for Fast‐Charging Solid‐State Batteries

**Figure 3:**
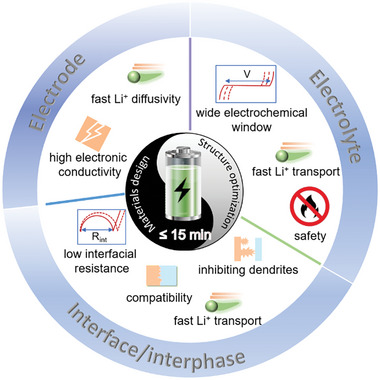
Schematic illustration of the key requirements for electrodes, electrolytes, and the electrode/electrolyte interface to enable fast‐charging SSBs.

The transport properties of SEs are crucial to achieving fast‐charging capabilities in SSBs. An ideal electrolyte for fast‐charging SSBs should exhibit high σ and a close‐to‐unity $t_{Li^{+}}$ to ensure rapid and efficient Li^+^ transport. Furthermore, it should demonstrate chemical compatibility with both anode and cathode materials and have a wide electrochemical window, preventing unwanted side reactions (**Figure** **3**). Additional critical requirements include thermal and mechanical stability, inherent safety, and environmental friendliness.^[^ ^12^, ^27^ ^]^ This section explores the ion transport mechanisms and recent achievements in ICEs, SPEs, and IPCs, examining their materials chemistry, structure, functional design, and applications in fast‐charging SSBs.

### 3.1 Inorganic Ceramic Electrolytes

**Figure 4:**
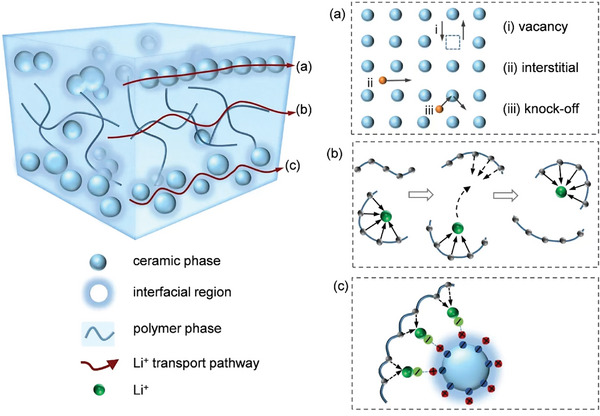
Schematics of ion transport mechanisms within SEs: a) Principal Li^+^ migration mechanisms of vacancy, interstitial, and interstitialcy in ICEs; b) Segmental motion of polymer chains; and c) Lewis acid‐base interactions between the ceramic filler and polymer interphase.

ICEs include sulfides (e.g., Li_10_GeP_2_S_12_), halides (e.g., Li_3_YCl_6_), and oxides (e.g., LLZO garnets, La_0.5_Li_0.5_TiO_3_ perovskites, NASICON).^[^ ^28^ ^]^ These ionic conductors exhibit better safety than commercial liquid electrolytes and possess a near‐unit $t_{Li^{+}}$, effectively minimizing concentration gradients at high current densities. Despite their advantages, most ICEs, generally except sulfides, face a significant challenge, insufficient ionic conductivity, which limits their potential for fast‐charging SSBs. This ionic conductivity is governed by Li⁺ diffusion pathways within the crystal structure, where ions migrate between specific crystallographic sites. Three primary ion hopping mechanisms have been identified:^[^ ^29^ ^]^ i) direct ion transport to adjacent vacant sites; ii) ion diffusion through interstitial sites between partially occupied lattice sites; and iii) knock‐off mechanisms, where interstitial ions displace lattice ions into neighboring sites (**Figure** **4**a).

**Figure 5:**
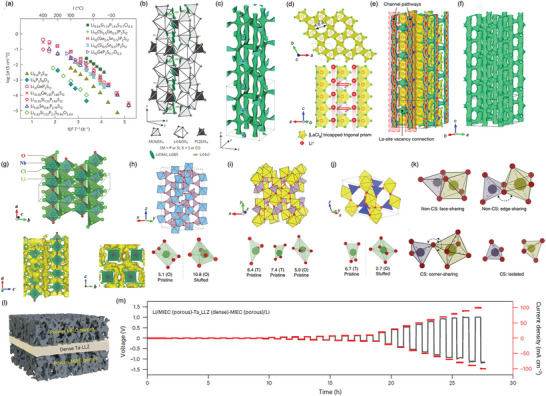
a) Arrhenius plots of ionic conductivities of Li_9.54_Si_1.74_P_1.44_S_11.7_Cl_0.3_, Li_9.6_P_3_S_12_, and the other specific materials. b) Crystal structure and c) Nuclear density distributions of Li atoms for Li_9.54_Si_1.74_P_1.44_S_11.7_Cl_0.3_. Panels a–c) Reproduced with permission.^[^ ^30^ ^]^ Copyright 2016, Springer. d) Structural model of a LaCl_3_‐lattice‐based Li^+^ superionic conductor, illustrating the Li^+^ migration mechanism. Li^+^ migrates along the 1D channel (red spheres) and between adjacent channels (bidirectional arrows; vacancies are represented by the gray tricapped trigonal prisms). e) Li^+^ probability density, represented by green isosurfaces from simulations at 900 K in the vacancy‐contained LaCl_3_ lattice. f) Isolated Li^+^ probability density isosurfaces through removal of all [LaCl_9_] polyhedrons to show the excellent interconnectivity of the 3D Li^+^ migration pathways. Panels d–f) Reproduced with permission.^[^ ^31^ ^]^ Copyright 2023, Springer. g) Crystal structure and Li^+^ probability density map for the superionic conductor LiNbOCl_4_ (LNOC). Reproduced with permission.^[^ ^32^ ^]^ Copyright 2023, Wiley‐VCH. h–k) Crystal structures of known superionic conductors with corner‐sharing frameworks. Reproduced with permission.^[^ ^33^ ^]^ Copyright 2022, Springer. l) Schematic representation of a trilayer architecture with porous mixed ion‐ and electron‐conducting garnet and dense Ta‐LLZO. m) CCD of a symmetric Li cell measured from 0.1 to 100 mA cm^−2^ at room temperature. Panels l–m) reproduced with permission.^[^ ^34^ ^]^ Copyright 2023, Springer.

Among ICEs, sulfides have the highest ionic conductivity (≈10^−2^ S cm^−1^), which is comparable to liquid electrolytes.^[^ ^16a^ ^]^ Furthermore, sulfides possess low grain boundary resistance and excellent malleability, enabling close electrode contact and facilitating cold‐press manufacturing. These characteristics make sulfides a compelling choice for fast‐charging SSBs. The Ceder group identified a body‐centered cubic (bcc)‐like anion framework as a structural motif that facilitates fast Li^+^ transport of sulfides. This facilitates Li^+^ hopping between neighboring sites and promotes direct jumps between adjacent tetrahedral sites, thereby contributing to higher ionic conductivity.^[^ ^35^ ^]^ Upon investigating the recently synthesized fast‐ion conductors, Li_10_GeP_2_S_12_ and Li_7_P_3_S_11_, the authors observed that the sulfur sublattices of both materials closely match a bcc lattice. This work systematically identified the compound attributes that lead to high Li^+^ conductivity, providing specific criteria for developing improved conductors. In 2016, Kato et al. developed a bcc‐type Li_9.54_Si_1.74_P_1.44_S_11.7_Cl_0.3_ with an exceptionally high ionic conductivity of 25 mS cm^−1^, surpassing previous sulfide‐based SEs (**Figure** **5**a).^[^ ^30^ ^]^ This high ionic conductivity stems from the 3D conduction pathways, as evidenced by the anisotropic thermal displacement of Li (Figure 5b) and nuclear density distribution (Figure 5c). The SSB with Li_9.54_Si_1.74_P_1.44_S_11.7_Cl_0.3_ demonstrated exceptional cycling performance with a high current density of 18C at 100 °C. Li et al. recently synthesized a series of halogen‐rich lithium argyrodites with the general formula of Li_5.5_PS_4.5_Cl*_x_* Br_1.5−_ *_x_* (0 ≤ *x* ≤ 1.5), demonstrating that increased S^2−^/Cl^−^/Br^−^ disorder quantified as configurational entropy, significantly accelerates Li^+^ dynamics.^[^ ^36^ ^]^ This structural complexity also increases vibrational entropy, enhancing ion diffusion through phonon–ion interactions.^[^ ^37^ ^]^ The maximum level of anion disorder was achieved in Li_5.5_PS_4.5_Cl_0.8_Br_0.7_, exhibiting a remarkable RT ionic conductivity of 22.7 mS cm^−1^.^[^ ^36^ ^]^

Sulfide‐based SEs, despite their superior ionic conductivity, face critical electrochemical stability challenges due to S^2−^ oxidation at 2–2.5 V.^[^ ^38^ ^]^ Interfacial byproducts in sulfide‐based SSBs increase charge‐transfer impedance and capacity loss. Furthermore, Ge, Si, Sn, and Sb, commonly used elements in conducting sulfides‐based SEs, are unstable against Li, narrowing the electrochemical windows. Incorporating halogens like I and Cl can aid in creating a protective SEI layer of Li halides, thereby impeding further reduction.^[^ ^39^ ^]^

Recently, lithium‐metal‐halide SEs designated as Li‐M‐X (where M = Y, Dy, Gd, Ho, La, Nd, Sc, Sm, Tb, or Tm, and X = F, Cl, Br, I, O, or S) have gained significant attention. Among these, chlorides and bromides generally exhibit wide electrochemical windows, low electronic conductivity, and good interfacial compatibility with cathode materials, making them desirable for SE applications.^[^ ^40^ ^]^ However, the ionic conductivity of halides, typically only a few mS cm^−1^, remains inferior to that of sulfides. This lower conductivity is primarily attributed to the close‐packed crystal structures of halides, which hinder facile Li^+^ migration. In 2023, to overcome this limitation, Yin et al. presented a superionic Li conductor (Li_0.388_Ta_0.238_La_0.475_Cl_3_) based on LaCl_3_ with an ionic conductivity of 3.02 mS cm^−1^ at 30 °C.^[^ ^31^ ^]^ Introducing La vacancies via Ta doping created large 1D channels forming a 3D network, facilitating rapid Li^+^ migration (Figure 5d–f). Similarly, Tanaka et al. reported lithium‐metal‐oxy‐halide materials, LiMOCl_4_, exhibiting high ionic conductivity of 10.4 mS cm^−1^ (M = Nb) and 12.4 mS cm^−1^ (M = Ta). These materials leverage the anion‐mixing of a divalent anion (O ion) with a halogen ion, along with high‐valent Nb and Ta cations, to form corner‐sharing polyhedral units, thereby enhancing potential Li^+^ migration pathways (Figure 5g).^[^ ^32^ ^]^ SSBs employing these oxyhalides as the cathode‐side SE and sulfides as the anode‐side SE demonstrated exceptional rate capability, maintaining 80% of their capacity at a high rate of 5C.

Amorphous lithium‐metal‐halide SEs have emerged as promising candidates for SSBs in recent years. Relative to their crystalline counterpart, amorphous SEs offer advantages, such as ease of synthesis, high ionic conductivity, and minimal grain boundary resistance.^[^ ^41^ ^]^ Zhang et al. synthesized a series of *x*Li_2_O‐TaCl_5_ (1 ≤ *x* ≤ 2) amorphous SEs, with 1.6Li_2_O‐TaCl_5_, achieving a RT ionic conductivity of 6.6 mS cm^−1^.^[^ ^42^ ^]^ A full cell utilizing this SE with a LiNi_0.83_Co_0.11_Mn0_.06_O_2_ cathode reached an initial capacity of 82 mAh g^−1^ at 3C, and demonstrated stable cycling of 2400 cycles at 2C with 90.7% capacity retention at 25 °C. Li et al. further reported a novel class of amorphous Li‐Ta‐Cl‐based chloride SEs, exhibiting high ionic conductivity (7.16 mS cm^−1^ at 25 °C) and low Young's modulus (3 GPa), thus facilitating not only efficient Li^+^ conduction but also good interfacial contact in SSBs.^[^ ^43^ ^]^ Consequently, SSBs incorporating these amorphous chloride SEs and a LiNi_0.88_Co_0.07_Mn_0.05_O_2_ cathode demonstrated remarkable capacity retention (≈99% after 800 cycles at 3C) and high‐rate capability (115.1 mAh g^−1^ at 4C). Furthermore, the battery exhibited exceptional long‐term cycling stability (≈77% capacity retention after 9800 cycles at ≈3.4C, even at −10 °C), highlighting the potential for fast‐charging SSBs.

For most lithium‐metal‐halide SEs, a potential key challenge lies in simultaneously achieving high ionic conductivity while maintaining environmental/electrochemical stability, particularly against Li. Therefore, advancing fast‐charging SSB technology demands the development of novel SEs that combine high ionic conductivity with electrode compatibility, underpinned by a thorough understanding of ion transport mechanisms and interfacial stability.

Oxide‐based SEs, such as garnets and NASICON‐type materials, offer excellent electrochemical stability and processability in air. Jun et al. demonstrated that a corner‐sharing framework provides access to a highly distorted Li environment and facilitates percolating pathways with low energy barriers.^[^ ^33^ ^]^ As illustrated in Figure 5h–j, a structural feature of known superionic conductors is the interconnection of non‐Li cation polyhedra by a corner‐shared oxygen, explicitly avoiding the sharing of edges (O*─*O bond) or faces (O*─*O*─*O triangle) (Figure 5k).^[^ ^33^ ^]^ One such example is the NASICON‐type compound LiGa(SeO_3_)_2_, which exhibits a bulk ionic conductivity of 0.11 mS cm^−1^ at RT.^[^ ^33^ ^]^ The ionic conductivity of oxide‐based SEs can be further increased through substitution, such as partially replacing Ti^4+^ in LiTi_2_(PO_4_)_3_ with M^3+^ (M = Al, Cr, Ga, Fe, Sc, In, Lu, Y, or La) and substituting a small amount of Ta, Al, Ga, Nb, or Te in LLZO to obtain a higher conducting cubic phase structure.^[^ ^44^ ^]^ Kim et al. tailored garnet‐type LLZO through bulk doping and interfacial protonation/etching, improving the CCD of LLZO from 0.6 mA cm^−2^ to 2.6 (Ta‐doped) and 2.0 mA cm^−2^ (Al‐doped) at 60 °C.^[^ ^45^ ^]^ Additionally, the use of a highly disordered amorphous Li‐garnet as a solid‐electrolyte separator layer in a microbattery has enabled cycling at a fast rate of 10C over 500 cycles while preventing Li dendrite formation with a 10 nm coating.^[^ ^46^ ^]^

Even though oxides are promising SEs, SSBs with pure oxides‐based SEs struggle to achieve satisfactory electrochemical performance at RT. These challenges stem from limited ionic conductivity, inadequate interfacial contact owing to their nonductility nature and high boundary resistance, ultimately compromising ion transport pathways.

The most recently reported ICEs with ionic conductivity, electrochemical oxidative stability, CCD, and the strategies for realizing fast‐charging SSBs are listed in **Table** **1**. Most ICEs are polycrystalline, in which they are composed of multiple single crystals, grain boundaries, isolated particles, impurities, and internal flaws. These internal factors crucially influence the internal ionic flux. Therefore, research should primarily focus on i) understanding and optimizing internal structures and compositions to enhance ionic conductivity and ii) discovering new fast Li^+^ conductors. Emerging materials such as medium‐entropy, amorphous Li garnets (e.g., amorphous LLZO),^[^ ^55^ ^]^ and high‐entropy Li argyrodites (e.g., Li_5.5_PS_4.5_Cl*_x_* Br_1.5−_ *_x_* (0 ≤ *x* ≤ 1.5))^[^ ^36^ ^]^ with superior ion transport demonstrate the potential for fast‐charging SSBs. Optimization of sintering processes, such as hot pressing, rapid sintering, and plasma sintering can enhance density and, consequently, improve ionic conductivity.^[^ ^49^, ^56^ ^]^ Wang et al. employed ultrafast high‐temperature sintering (UHS) to produce high‐density (≈97%) LLZO with a small grain size (8.5 ± 2 µm), achieving a CCD of 3.2 mA cm^−2^ in a Li|LLZO|Li symmetric cell.^[^ ^49^ ^]^ Modifications to particle size and structure have demonstrated the potential for improving interfacial contact and electrochemical performance of ICE‐based SSBs. Wu and co‐workers used freeze‐drying to synthesize Li_3_InCl_6_ nanoparticles, reducing the roughness of the SE layer interface, and effectively minimizing the contact gap between interfaces.^[^ ^54^ ^]^ As a result, an SSB incorporating a double‐layer electrolyte (sulfide‐based anolyte and small‐particle halide‐based catholyte), along with a LiNi_0.9_Co_0.05_Mn_0.05_O_2_ cathode and a Li anode, delivered long operational stability of 30 000 cycles at a high current rate of 20C. Alexander et al. recently developed a trilayer architecture with a porous mixed ion‐ and electron‐conducting (MIEC) framework supporting a 15 µm‐thin Ta‐doped LLZO electrolyte (Figure 5l), achieving an unprecedented CCD of 100 mA cm^−2^ (Figure 5m).^[^ ^34^ ^]^ **Table** **2** summarizes recently developed high‐rate (≥4 C) SSBs with operation temperature, battery configurations, electrochemical performance, and strategies. The multilayer design with high ion conductive ICEs is promising. Nevertheless, compared to commercial separators with liquid electrolyte, the larger thickness of ICEs increases the Li^+^ migration distance, reduces Li^+^ conduction, and limits the energy and power density of SSBs. To this end, many recent works introduce new methods for preparing thin ICEs such as printing and fast sintering process, solvent‐free procedure and tape casting method.^[^ ^57^ ^]^ The development of ultrathin and high‐conductive ICEs can shorten the Li^+^ conduction pathway in a full battery, and thus more attention should be paid to this aspect in the future.

**Table 1:** Summary of ionic conductivity, electrochemical oxidative stability, and CCD of recently reported ICEs for fast‐charging SSBs.

ICE type	ICE formula/component	σ [mS cm^−1^] @temperature	Oxidative stability	CCD [mA cm^−2^] @temperature	Strategy	Refs.
Sulfides	Li_9.54_Si_1.74_P_1.44_S_11.7_Cl_0.3_ (LSiPSCl)	25@RT	2.6 V	–	Formation of 3D conduction pathways	[30]
	Li_3_PS_4_‐Li_4_SiS_4_	2.21@25 °C	–	1.2@25 °C	Engineering an Li_3_PS_4_‐Li_4_SiS_4_ complex structure within a sulfide glass network	[39]
	LiF@Li_10_GeP_2_S_12_	2.54@25 °C	≈4.6 V	3.0@25 °C	Formation of LiF‐coated core–shell SEs	[47]
	Li_5.5_PS_4.5_Cl_1.5_	9.03@RT	–	–	Tailoring the composition to obtain chlorine‐rich argyrodite electrolytes	[48]
Halides	Li_0.388_Ta_0.238_La_0.475_Cl_3_	3.02@30 °C	4.35 V	5.0@RT	Ta doping, creating large 1D channels	[31]
	LiNbOCl_4_	10.4@RT	5 V	–	Formation of corner‐sharing polyhedral units	[32]
	1.6Li_2_O‐TaCl_5_	6.60@25 °C	4.15 V	–	Amorphous structure to reduce grain boundary resistance	[42]
Oxides	LLZO	1.00@25 °C	≈3.0 V by DFT	3.2@25 °C	Ultrafast high‐temperature sintering process	[49]
	Ta‐LLZO	0.51@25 °C	–	2.6@60 °C	Bulk doping and interfacial protonation/etching	[45]
	Al‐LLZO	0.29@25 °C		2.0@60 °C		
	Ta‐LLZO	0.44@25 °C	≈6.0 V	1.6@25 °C	Interlayer strategy with a Ag‐coated Ta‐LLZO and Ag‐C composite interlayer	[50]
	Ta‐LLZO	0.23@RT	–	100.0@RT	Formation of a MIEC garnet 3D architecture	[34]

**Table 2:** Summary of electrochemical performance of high‐rate (≥4 C) SSBs with respect to operation temperature, key battery components and parameters and strategies.

Anode active material	Cathode active material	Complex cathode composition [wt%]	Cathode mass loading	Solid electrolyte	Operation temperature	Capacity/C‐rate/cycle	Strategy	Refs.
Li_4_Ti_5_O_12_	LiCoO_2_	LiNbO_3_‐coated LiCoO_2_:LSiPSCl:Acetylene black = 60:34:6	–	LSiPSCl	100 °C	82 mAh g^−1^ /18C/500	Introduction of sulfide superionic conductors	[30]
Li‐graphite	Single‐crystal LiNi_0.8_Mn_0.1_Co_0.1_O_2_ (NMC811)	NMC811:Li_6_PS_5_Cl:PTFE = 67.9:29.1:3	2 mg cm^−2^	Li_6_PS_5_Cl/ Li_10_GeP_2_S_12_/ Li_6_PS_5_Cl	55 °C	81 mAh g^−1^ /20C/10 000	Multilayer electrolyte design	[16b]
In‐Li	LiNi_0.6_Mn_0.2_Co_0.2_O_2_ (NMC622)	NMC622: Li_5.5_PS_4.5_Cl_1.5_ = 70:30	2.5 mg cm^−2^	Li_5.5_PS_4.5_Cl_1.5_	RT °C	62.3 mAh g^−1^/10C/10 000; 102.2 mAh g^−1^ /5C/4500	Introduction of chlorine‐rich argyrodite electrolytes	[48]
In‐Li	Single‐crystalline LiNi_0.88_Co_0.11_Al_0.01_O_2_ (NCA)	NCA:Li_3_YCl_6_:super C65 = 70:50:3 weight ratio	11.3 mg cm^−2^	Li_6_PS_5_Cl_0.5_Br_0.5_	30 °C	130 mAh g^−1^/4C/NA	Introduction of single crystalline cathode and halide electrolyte	[51]
Li‐graphite	LiNi_0.83_Mn_0.06_Co_0.11_O_2_	LiNi_0.83_Co_0.11_Mn_0.06_O_2_:Li_5.5_PS_4.5_Cl_1.5_:PTFE = 70:30:3 weight ratio	2 mg cm^−2^	Li_5.5_PS_4.5_Cl_1.5_/ Li_6_PS_5_Cl/ Li_5.5_PS_4.5_Cl_1.5_	55 °C	128 mAh g^−1^/20C/700	Introduction of chlorine substituted argyrodite electrolyte	[52]
In	NMC622	NMC622:Li_3_N:Li_6_PS_5_Cl:graphite hollow nanocarbon = 71.25:3.75:23:2	3.25 mg cm^−2^	Li_6_PS_5_Cl	55 °C	50 mAh g^−1^/4C/NA	Introduction of Li_3_N sacrificial cathode	[53]
Graphite	LiCoO_2_	LiCoO_2_:LiNbOCl_4_ = 82.7:17.3	–	LiNbOCl_4_/ Li_6_PS_5_Cl	25 °C	96 mAh g^−1^/5C/NA	Introduction of lithium‐metal‐oxy‐halide materials with high ionic conductivity and oxidation stability	[32]
Li	LiNi_0.9_Mn_0.05_Co_0.05_O_2_ (NMC90)	NMC90:Li_3_InCl_6_ = 8:2	9.8 mAh cm^−2^	Li_3_InCl_6_/ Li_6_PS_5_Cl	25 °C	17 mAh g^−1^/49C; 60 mAh g^−1^/49C/30 000	Freeze‐drying technology	[54]
Li	NMC622	–	2.3 mAh cm^−2^	MIEC/Ta‐LLZO/MIEC	25 °C	80 mAh g^−1^/6.9 mA cm^−2/NA^	Development of a single‐phase MIEC garnet	[34]

### 3.2 Solid Polymer Electrolytes

SPEs offer several advantages over ICEs, including better flexibility, improved interfacial adhesion, and easier processability.^[^ ^58^ ^]^ Commonly used polymer matrix materials for SPEs include polyethylene oxide (PEO), poly(methyl methacrylate) (PMMA), poly(vinylene carbonate) (PVCA), polyacrylonitrile (PAN), polyvinylidene fluoride (PVDF), and poly(vinylidene fluoride‐hexafluoropropylene) (PVDF‐HFP).^[^ ^59^ ^]^ However, the high degree of crystallization exhibited by most polymer chains at RT limits ion transport, as it is primarily facilitated by segmental motion in the amorphous regions (Figure 4b), thus resulting in low RT ionic conductivity.^[^ ^12b^ ^]^ Additionally, the $t_{Li^{+}}$ in SPEs is typically low (≈0.2–0.5) because both the Li^+^ and anions are mobile.^[^ ^60^ ^]^ Furthermore, for polymers such as the commonly used PEO, the inclusion of terminal hydroxide group (−OH) in the polymer poses a restriction on their suitability with high‐voltage cathode and Li anode, thus limiting their electrochemical stability window.^[^ ^61^ ^]^ Consequently, to realize fast‐charging SSBs based on SPEs, considerable research has focused on regulating the Li^+^ transport channel to enhance both σ and $t_{Li^{+}}$, and enlarge the electrochemical window.

**Figure 6:**
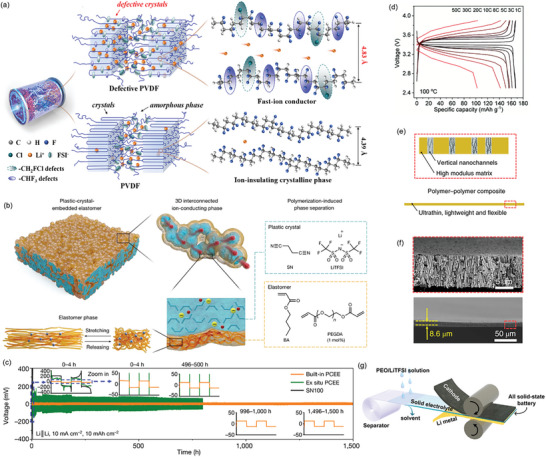
a) Schematic of Li^+^ transport in defective PVDF and traditional PVDF crystals. Reproduced with permission.^[^ ^62^ ^]^ Copyright 2024, The Royal Society of Chemistry. b) Design and structure of the plastic‐crystal‐embedded elastomer electrolyte. c) Li plating and stripping performance at 10 mA cm^−2^ with various polymer electrolytes, highlighting the in situ formed elastomeric electrolyte (orange line). Panels b,c) Reproduced with permission.^[^ ^63^ ^]^ Copyright 2022, Springer. d) Typical charge/discharge curves at various C rates of a Li|S‐LHCE|LiFePO_4_ battery. Reproduced with permission.^[^ ^64^ ^]^ Copyright 2022, The Royal Society of Chemistry. e) Schematic illustration of the design principles for polymer–polymer composite electrolytes featuring a high‐modulus matrix with vertical nanochannels for enhanced ionic transport. f) Cross‐sectional SEM images of an ultrathin nanoporous PI film (bottom) with a zoomed‐in view of the aligned nanopores (top). Panels e,f) Reproduced with permission.^[^ ^59a^ ^]^ Copyright 2019, Springer. g) Schematic representation of the assembly process for SSBs incorporating a PPL electrolyte, consisting of a PE separator infused with a PEO/LiTFSI electrolyte solution. Reproduced with permission.^[^ ^65^ ^]^ Copyright 2019 Wiley‐VCH.

Because of the tight chain packing, Li^+^ migration is impeded in polymeric crystals. However, the Bruce group developed a series of PEO_6_:LiXF_6_ (X = P, As, and Sb) SPEs, illustrating that clusters of PEO chains fold into cylindrical tunnels, facilitating the movement of Li^+^ ions between sites independently of segmental motion.^[^ ^14^ ^]^ This discovery has encouraged the modification of crystal structure. Recently, Dai et al. proposed for the first time to introduce trifluoroethylene and chlorofluoroethylene monomers into the VDF crystals as dipolar defects, thereby transforming the original ion‐insulating PVDF crystalline phase into a fast ion conductor (**Figure** **6**a).^[^ ^62^ ^]^ The developed PVDF‐based SPE had an extremely high RT ionic conductivity of 0.78 mS cm^−1^, enabling the SPE‐based Li/LiFePO_4_ to operate at 5C while retaining ≈100% capacity after 400 cycles. The incorporation of plastic crystals like succinonitrile (SN) that exhibits plastic‐crystal behavior across a wide temperature range (−35 to 62 °C) and effectively solvates Li salts due to its high polarity, offers a promising path to enhance the ionic conductivity of SPEs.^[^ ^66^ ^]^ For example, Lee et al. developed an in situ‐formed elastomeric electrolyte using a 3D interconnected SN by chemically cross‐linking butyl acrylate and poly(ethylene glycol) diacrylate as the elastomer networks (Figure 6b), thus combining the benefits of both elastomer and plastic crystal.^[^ ^63^ ^]^ The electrolyte exhibited high ionic conductivity (1.1 mS cm^−1^ at 20 °C), high Li^+^ transference number ($t_{Li^{+}}$= 0.75), good mechanical robustness (300% tensile strain), a wide electrochemical window (> 4.6 V), and low interfacial resistance (175 Ω cm^2^ for 30 days against Li). The symmetric Li battery with elastomeric electrolyte had stable cycling performance at 10 mA cm^−2^ (Figure 6c).

Solvent‐soaked polymer membranes, known as quasisolid polymer electrolytes (QSPEs), though not strictly classified as SEs, have emerged as promising alternatives. These porous membranes, infused with liquid solvents, enable Li⁺ to transport through the liquid and swollen, gelled phase, as well as through the segmental motion of polymer chains. This multimodal transport mechanism often yields ionic conductivities surpassing those of SPEs and approaching those of liquid electrolytes.^[^ ^67^ ^]^ Xu et al. developed a solidified local high‐concentration electrolyte (S‐LHCE) via the freeze‐drying method.^[^ ^64^ ^]^ This S‐LHCE exhibited enhanced ionic conductivity (0.27 mS cm^−1^), high $t_{Li^{+}}$ i = 0.72), and improved electrode/electrolyte interface compatibility due to decoupled ion pairing and transport. The Li|S‐LHCE|LiFePO_4_ batteries demonstrated a wide operational temperature range (−10 to 100 °C) and remarkable capacity retention (83.3% and 60.1% of the theoretical capacity at 30C and 50C, respectively (Figure 6d). Li et al. developed a high conductive QSPE via in situ polymerization using a 1,3,5‐trioxane monomer, and a low‐melting‐point, modest‐viscosity 2,2,2‐Trifluoro‐*N*, *N*‐dimethylacetamide solvent. The QSPE‐based Li/NMC811 battery demonstrated stable capacities across different C‐rates (e.g., ≈198 mAh g^−1^ at 0.5C and 118 mAh g^−1^ at 10C).^[^ ^68^ ^]^ We developed a series of QSPEs by optimizing Li salt‐solvent‐polymer interactions to achieve high electrochemical performance. For instance, through the rational formulation of electrolyte ingredients, a unique multilayer solvation structure of in situ QSPE with high ionic conductivity of 1.0 mS cm^−1^ at −30 °C was obtained, demonstrating the potential of fast‐charging applications.^[^ ^69^ ^]^ By polymerizing in situ trifluoroethyl methacrylate in fluorinated ethylene carbonate, an all‐fluorinated QSPE with expanded electrochemical window of 4.89 V, high ionic conductivity of 1.88 mS cm^−1^ at RT, and nonflammability was developed, enabling SSBs with a stable cycling performance at 5C under a high cut‐off voltage of 4.5 V.^[^ ^70^ ^]^ We also reported a safe and single‐ion conductive QSPE by adjusting the Li^+^ solvation through a weak interaction with the polymer skeleton and strong coordination with NO^3−^, obtaining a record‐high rate capability among QSPE‐based SSBs, with a power density of 789 W kg^−1^.^[^ ^71^ ^]^ Similarly, Pei et al. developed a nonflammable, Li−N interaction induced 1,2‐dimethylimidazole‐based deep eutectic polymer electrolyte with high ionic conductivity (1.67 mS cm^−1^) and $t_{Li^{+}}$ (0.65), resulting in high reversible capacities even at 10C.^[^ ^72^ ^]^

Reducing the thickness of SPEs can shorten the Li^+^ transport path, potentially improving both energy density and fast‐charging performance. For example, the Cui group developed an SPE consisting of a flexible, nonflammable, porous polyimide (PI) host with PEO/LiTFSI fillers.^[^ ^59a^ ^]^ The 8.6 µm thick PI matrix, featuring vertically aligned nanochannels (Figure 6e,f), exhibited higher ionic conductivity (0.23 mS cm^−1^ at 30 °C) compared to a PEO/LiTFSI thin film (5.4 × 10^−2^ mS cm^−1^). We presented a nonflammable and dual‐salt SPE with porous polytetrafluoroethylene scaffold by tape casting. The proposed SPE has an ultralow thickness of 20 µm, high ionic conductivity of 0.45 mS cm^−1^ at RT, and a wide electrochemical window of 4.91 V, improving the cycling stability and rate performance of Li/NMC811 batteries with a discharge capacity of 112 mAh g^−1^ over 1000 cycles at 2C and RT.^[^ ^73^ ^]^ Similarly, Wu et al. developed a 7.5 µm‐thick SPE named PPL by integrating PEO/LiTFSI into a polyethylene separator (Figure 6g).^[^ ^65^ ^]^ The ultrathin PPL effectively shortened Li^+^ diffusion time and distance within the electrolyte, resulting in Li|PPL|LiFePO_4_ batteries with an initial capacity of 135 mAh g^−1^ at RT and high‐rate capacities up to 10C at 60 °C.

**Table** **3** summarizes the properties of recently reported SPEs (not strict limited to SEs) that enable high charge rates. Efforts are still needed to enhance SPE performance for fast‐charging SSBs by overcoming initially low RT ionic conductivity, limited $t_{Li^{+}}$, and poor electrochemical and thermal stability. Polymer engineering techniques such as copolymerization, cross‐linking, and grafting can be employed to improve ionic conductivity, enhance electrochemical stability, and so on.^[^ ^11^, ^82^ ^]^ Additionally, increasing $t_{Li^{+}}$ is crucial in reducing polarization resistance and preventing Li dendrite growth. This can be achieved by incorporating suitable additives or functional groups that favor Li^+^ transport while blocking the counterion.^[^ ^83^ ^]^ Furthermore, incorporating additional functionalities, such as self‐healing, overheating protection, and self‐extinguishing features, will benefit thermal stability and safety during fast charging.^[^ ^84^ ^]^

**Table 3:** Summary of recently reported SPEs, IPCs, and QSPEs for fast‐charging SSBs.

Electrolyte	σ [mS cm^−1^] @temperature	$t_{Li^{+}}$ [RT]	Oxidative stability [V]	Cathode active material	Capacity [mAh g^−1^]/C‐rate [C]	Strategy	Operation temperature [℃]	Refs.
S‐LHCE	0.27@RT	0.72	5.0	LiFePO_4_	141.6/30C; 102.2/50C; 151.3/20C	Employment of a solidified localized high‐concentration electrolyte	100	[64]
1,3,5‐trioxane‐based QSPE	0.22@−20 °C	0.80 (−20 °C)	5.6	NMC811	150/5; 130/8; 118/10	Tailoring QSPE with in situ polymerization using a 1,3,5‐trioxane‐based precursor	30	[68]
Poly(butyl acrylate)‐based QSPE	2.1@RT	0.86	4.6	LiFePO_4_	122/5	Adjustment of the Li^+^ solvation through a weak interaction of QSPE	25	[71]
Deep eutectic polymer electrolyte	1.67@30 °C	0.65 (30 °C)	4.35	LiFePO_4_	79/5; 39/10	Formation of a deep eutectic electrolyte induced by Li−N interaction	25	[72]
PolyDOL with 10 wt% Ta‐LLZO	0.47@RT	0.78	5.1	LiFePO_4_	118.3/5C	Elimination of the space charge layer at the organic/inorganic interfaces	25	[74]
PVDF with Li_1.4_Al_0.4_Ti_1.6_(PO_4_)_3_	0.7@RT	0.67	4.77	NMC811	130/5	Construction of a weak‐interaction environment of electrolyte	25 °C	[75]
PVDF‐Li_3_Zr_2_Si_2_PO_12_‐ionic liquids	0.83@RT	0.81	5.01	LiFePO_4_	133.2/5; 111.1/10	Introduction of polymer‐compatible ionic liquids to mediate between ceramics and the polymer matrix	25	[76]
PVFH‐PVCA	2.04@RT	0.61	≈5.3	Li_1.2_Mn_0.56_Ni_0.16_Co_0.08_O_2_	141.9/4; 126.8/6; 109.2/8	Fabrication of an entanglement association polymer electrolyte	25	[77]
Polyimides (PI)‐PVDF	0.186@30 °C	0.42	4.5	LiFePO_4_	117.2/4	Development of a PI‐reinforced PVDF‐based polymer electrolyte	25	[78]
MOF‐TOf	1.1@RT	0.57	4.6	LiFePO_4_	117/5; 80/20	Introduction of a fluorinated MOF	25 °C	[79]
ZIF‐67‐LA‐PAM	3.84@30 °C	0.627 (30 °C)	5.2	LiFePO_4_	102.7/10; 72.2/20	Preparation of MOF‐natural polymer composite electrolyte by electrospinning	30	[80]
PVAC/TMS‐based CPE	0.48@RT	0.48	4.8	LiCoO_2_	142/4C	Development of a selectively wetted rigid‐flexible coupling strategy	25 °C	[81]

### 3.3 Inorganic Polymer Composite Electrolytes

**Figure 7:**
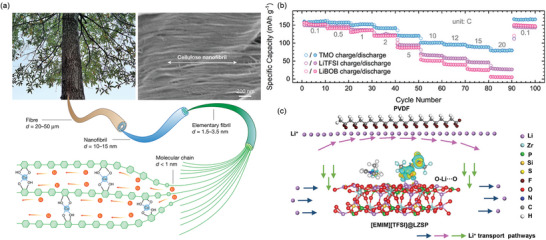
a) Schematic illustration of the hierarchical structure of cellulose nanofibrils (CNFs). Reproduced with permission.^[^ ^88^ ^]^ Copyright 2021, Springer. b) Rate performance of Li/LiFePO_4_ batteries with the LiTFSI@MOF‐OTf (TMO) electrolyte compared to those with liquid electrolytes (LiBOB and LiTFSI). Reproduced with permission.^[^ ^79^ ^]^ Copyright 2024, Wiley‐VCH. c) Schematic diagram of the Li^+^ transport pathways through ceramics, polymer matrix, and activated ceramic/polymer interphase. Reproduced with permission.^[^ ^76^ ^]^ Copyright 2024, Wiley‐VCH.

IPCs, which are conventionally constructed with a polymer matrix (e.g., PEO, PAN, PVDF, etc.) and inorganic fillers (either Li‐insulating fillers or ionic conductive fillers), generally exhibit enhanced σ and $t_{Li^{+}}$ compared to SPEs. IPCs can be either “ceramic‐in‐polymer” or “polymer‐in‐ceramic”, depending on the relative amount of ceramic fillers and polymers. The ceramic‐in‐polymer‐based IPCs, known for excellent mechanical flexibility and processability, can be fabricated via common approaches including tape casting, in situ polymerization, melt blending, and electrospinning,^[^ ^85^ ^]^ while polymer‐in‐ceramic‐based IPCs are usually prepared by hot/cold pressing techniques.^[^ ^12^, ^86^ ^]^ Common nonionic conductive inorganic fillers, including Al_2_O_3_, SiO_2_, TiO_2_, MgO, ZrO, and BaTiO_3_,^[^ ^85^, ^87^ ^]^ and other nonconductive fillers, such as cellulose and metal–organic frameworks (MOFs), can also enhance ionic conductivity and mechanical strength. The advantages of ceramic‐in‐polymer‐based IPCs include factors, such as reduced glass transition temperature (*T*_g_) and Lewis acid‐base interactions among the inorganic filler, polymer matrix, and Li salt (Figure 4c).^[^ ^11^, ^12^ ^]^ The interaction promotes segmental motion and salt dissociation, thus increasing Li^+^ mobility. Additionally, space‐charge layers at filler‐polymer interphases have been reported to facilitate cation transport.^[^ ^12^, ^25^ ^]^ For instance, the Hu group demonstrated rapid Li^+^ transport along polymer chains using Cu^2+^‐coordinated 1D amorphous Li‐conducting cellulose, which effectively opened the polar functional groups in the cellulose molecular channels (**Figure** **7**a), exhibiting high ionic conductivity of 1.5 mS cm^−1^ and a high $t_{Li^{+}}$ of 0.78.^[^ ^88^ ^]^ Wang et al. introduced a fluorinated MOF with triflate groups as a porous solid host. This design facilitated the decoupling of internal Li^+^ and provided hopping sites for fast Li^+^ transport, resulting in a high RT ionic conductivity of 1.1 mS cm^−1^ and a high $t_{Li^{+}}$ of 0.57.^[^ ^79^ ^]^ The SSBs with fluorinated MOF‐based electrolyte (MOF‐OTf) delivered excellent rate performance from 0.1C to 20C (Figure 7b). Similarly, Guan et al. integrated an in situ composite MOF (ZIF‐67) into a lithium alginate (LA) and polyacrylamides (PAM) membrane.^[^ ^80^ ^]^ The IPC showed enhanced mechanical strength and facilitated Li^+^ transport channels through hydrogen bonding interaction between MOF and LA‐PAM matrix.The IPC‐based symmetric Li cells exhibited superior stable plating and stripping performance at 40 and 100 mA cm^−2^, and the Li|ZIF‐67‐LA‐PAM|LiFePO_4_ batteries delivered long‐term cycling stability at 10C and 20C.

Li^+^ conductive inorganic fillers, primarily garnets (e.g., LLZO), perovskites (e.g., La_0.5_Li_0.5_TiO_3_), NASICON‐type materials (e.g., Li_1.5_Al_0.5_Ge_1.5_(PO_4_)_3_), and sulfides (e.g., Li_10_GeP_2_S_12_), offer a promising avenue for enhancing the performance of SPEs and QSPEs.^[^ ^28^, ^89^ ^]^ Incorporating these fillers into polymer electrolytes combines the advantages of both materials, namely the high ionic conductivity and mechanical strength of ceramics with the favorable interfacial properties and flexibility of the polymers. This synergy can lead to improved cycling stability and rate performance in SSBs. The Cui group exemplified this approach by designing an IPC comprising PVDF, polyvinyl acetate (PVAC), Li_6.4_La_3_Zr_1.4_Ta_0.6_O_12_ (LLZTO), and tetramethylene sulfone (TMS).^[^ ^81^ ^]^ The rigid PVDF/LLZTO component provided mechanical strength, while the flexible PVAC/TMS component offered high ionic conductivity and a wide electrochemical window. The fabricated IPC had a high ionic conductivity of 0.48 mS cm^−1^, a high $t_{Li^{+}}$ of 0.48, and compatibility with a high‐voltage LiCoO_2_ cathode. The developed IPC based Li/LiCoO_2_ battery exhibited a discharge capacity of 142 mAh g^−1^, even at a high rate of 4C.

Reducing the size of ceramic fillers to the nanoscale generally enhances the ionic conductivity of IPCs by increasing the interfacial area between the filler and the polymer matrix. This interfacial area can build a space charge region that may provide a Li^+^ fast conduction pathway.^[^ ^90^ ^]^ The increased interfacial area may also enhance Lewis acid–base interactions between the filler surface and polymer chains, hinder the crystallization of polymer chains, and enhance salt dissociation.^[^ ^12b^ ^]^ Liu et al. investigated the polymer/ceramic interphase in IPCs using solid‐state nuclear magnetic resonance spectroscopy, revealing that optimizing the local interphase environment between the inorganic and polymer components is a promising avenue for designing IPCs with significantly improved conductivity.^[^ ^91^ ^]^ Similarly, Zhu et al. demonstrated that introducing polymer‐compatible ionic liquids between ceramics and the polymer matrix activates the ceramic/polymer interphase.^[^ ^76^ ^]^ This creates interpenetrating channels that promote efficient Li^+^ transport (Figure 7c), as evidenced by the exceptional rate performance of the composite solid electrolyte‐based Li/NMC811 battery, which sustained a capacity of 111.1 mAh g^−1^ even at a high rate of 10C. These studies underscore the importance of tailoring the ceramic/polymer interphase to increase Li^+^ transport pathways, ultimately enhancing the ionic conductivity of IPCs.

IPCs, which incorporate ceramic fillers as reinforcing components, have the potential to achieve high ionic conductivity. However, challenges such as low chemical compatibility and limited physical contact between the ceramic and polymer phases can hinder the formation of effective ion‐conducting networks.^[^ ^90^, ^92^ ^]^ Additionally, filler aggregation within IPCs impede ion migration.^[^ ^87^ ^]^ These challenges can be addressed by constructing unique ceramic microstructures, such as 1D nanowires,^[^ ^93^ ^]^ 2D nanosheets,^[^ ^94^ ^]^ and 3D frameworks^[^ ^95^ ^]^ that mitigate agglomeration while enhancing ionic conductivity and $t_{Li^{+}}$. Moreover, implementing chemical crosslinking between fillers and polymers improves interfacial contact, creating Li^+^ transport pathways along the filler‐polymer interphase and improving ionic conductivity.^[^ ^34^ ^]^ Surface modification of fillers, particularly Li^+^ conductors,^[^ ^96^ ^]^ facilitates better filler dispersion within the polymer matrix, enabling efficient Li^+^ transport through the ceramic phase.

## 4 Electrodes for Fast‐Charging Solid‐State Batteries

Optimizing electrode materials plays a critical role in addressing fast‐charging challenges. Commercial LIBs commonly use graphite anodes, which face fast‐charging limitations due to slow intercalation, increased electrode polarization, and Li plating reaction. These issues can lead to capacity fade and safety concerns. Additionally, the mechanical stress induced by the rapid volume changes during fast charging can cause graphite particle cracking and pulverization, leading to loss of electrical contact and further capacity degradation.^[^ ^9a^ ^]^ Li_4_Ti_5_O_12_ can effectively prevent Li plating thanks to a high operating potential of around 1.55 V (vs Li/Li^+^),^[^ ^97^ ^]^ but its application in SSBs has been limited due to compatibility issues with SEs, such as the formation of high‐resistance interphases and a lower energy density compared to graphite.^[^ ^98^ ^]^ For SSBs, alternative high‐capacity anodes, such as Li and micrometer‐scale Si, offer potential solutions under fast‐charging conditions.^[^ ^99^ ^]^ Li metal anodes have high theoretical capacity and low redox potential, but they face challenges related to dendritic growth and unstable solid electrolyte interface (SEI) formation during fast charging.^[^ ^100^ ^]^ Si anodes have high theoretical capacity but suffer from severe volume changes during lithiation/delithiation, leading to mechanical degradation and poor cycling stability, especially under fast‐charging conditions.^[^ ^101^ ^]^ On the cathode side, transition‐metal cations mediate charge gain and loss during Li^+^ insertion and extraction. Fast charging is limited by the Li^+^ diffusion within the cathode particles' lattice and Li^+^ transfer at the cathode‐electrolyte interface (CEI). Furthermore, the mechanical stresses linked to rapid Li^+^ insertion/extraction can lead to particle cracking and to structural degradation, eventually hindering long‐term cycling stability. Enhancing ion and electron transport within the cathode material by maximizing ion diffusivity and electronic conductivity is essential.^[^ ^102^ ^]^ The following section primarily focuses on two key factors that influence electrode kinetics: the intrinsic properties of electrode active materials and electrode microstructures.

### 4.1 Alternative Anode Active Materials

Li^+^ transport into anode active materials, such as intercalation (graphite and Li_4_Ti_5_O_12_), alloying (Si and Sn), or deposition as metal, is a significant limiting factor for fast‐charging SSBs.^[^ ^103^ ^]^ To be capable of fast charging, the active material should possess a low barrier for Li^+^ transfer and fast bulk solid‐state diffusion. In contrast to intercalation‐type materials, high‐capacity alloy‐type anodes allow for reduced electrode thickness and charge transport distance, which positively impact overall fast‐charging performance, especially when paired with SEs that do not penetrate the porous electrode. However, the substantial volume expansion of common alloy‐type anodes can induce uncontrolled morphological changes,^[^ ^104^ ^]^ posing a significant challenge to fast‐charging SSBs.

**Figure 8:**
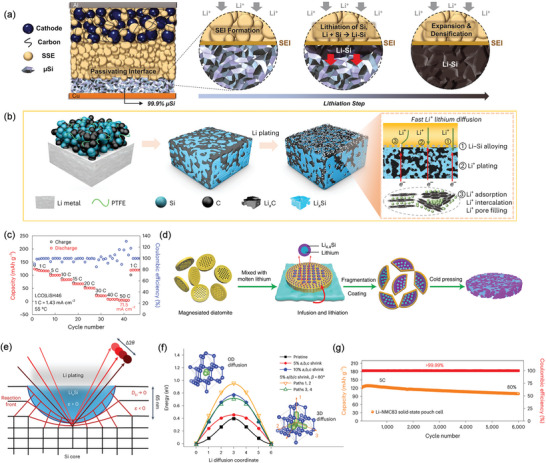
a) Schematics of the µSi electrodes within SSBs. Reproduced with permission.^[^ ^105^ ^]^ Copyright 2021, The American Association for the Advancement of Science. b) Schematics of the hard‐carbon‐stabilized Li‐Si anode. c) Rate performance of LiSH46|Li_6_PS_5_Cl|LiCoO_2_ batteries at 55 °C. Panels b,c) Reproduced with permission.^[^ ^106^ ^]^ Copyright 2023, Springer. d) Schematic illustration of the fabrication process for the constructed hierarchical Si‐Li based composite anode with PEO‐SPE. Reproduced with permission.^[^ ^107^ ^]^ Copyright 2019, Springer. e) Illustration of “constriction susceptibility” in anode materials and the diffusion‐limiting process during Li plating. The red region represents the reaction front, where compressive strain induces a significant reduction in Li diffusivity (*D*_Li_), effectively approaching zero. As a result, Li plating is kinetically favored at these specific surface nanosites. The effect also causes strain broadening (Δ2θ) for Si XRD peaks. f) Diffusion pathway of Li^+^ within the Si unit cell. g) Cycling performance of a solid‐state pouch cell with a LiNi_0.83_Mn_0.06_Co_0.11_O_2_ cathode (15 mg cm^−2^) at 5C, 55 °C, and 25 MPa. Panels e,–g) Reproduced with permission.^[^ ^108^ ^]^ Copyright 2024, Springer.

To better accommodate volume changes, Tan et al. created micrometer‐scale silicon particles (µSi).^[^ ^105^ ^]^ As shown in **Figure** **8**a, the SE cannot permeate through the porous µSi electrode, reducing the interfacial contact area to a 2D plane. During µSi lithiation, the formation of Li–Si can propagate throughout the electrode, benefiting from the direct ionic and electronic contact between Li–Si and µSi particles. After µSi lithiation, the 2D plane remains unchanged despite volume expansion, effectively preventing the generation of new interfaces. As a result, the µSi/NMC811 with a sulfide SE delivers a capacity retention of 80% after 500 cycles at 5 mA cm^−2^. Miyazaki et al. developed an amorphous Si film anode, showing good cycling performance even at a high rate of 10 mA cm^−2^ (i.e., 200C for a 50‐nm‐thick film).^[^ ^109^ ^]^ The Wu group developed a hard‐carbon‐stabilized Li‐Si alloy anode for SSBs. During cycling, a 3D Li^+^‐electronic conducting network, which contained Li_15_Si_4_ and LiC_6_ was built (Figure 8b), improving Li^+^ transport and providing fast electrode kinetics.^[^ ^106^ ^]^ The SSB, using the hard‐carbon‐stabilized Li–Si alloy anode (LiSH46), LiCoO_2_ cathode, and Li_6_PS_5_Cl electrolyte functioned without short circuits even at the ultrahigh rate of 50C and 55 °C (Figure 8c), and delivered favorable cycling stability over 30 000 cycles with a capacity retention of 72% at 20C. Zhou et al. constructed a hierarchical Si‐Li based composite anode with PEO‐based SPE by cold pressing (Figure 8d).^[^ ^107^ ^]^ This construction enhanced electrode/electrolyte contact, decreased interface resistance, and consequently boosted the rate performance of SSBs, achieving a high rate capability of 65 mAh g^−1^ at 5C.

Ye et al. recently unveiled a new phenomenon of “constriction susceptibility” at the solid–solid interface between Li and Si in SSBs.^[^ ^108^ ^]^ This refers to the confinement of the lithiation reaction in micrometer‐sized Si particles to thin surface sites, rather than extensive Li–Si alloying throughout the particles, as typically observed in conventional solid–liquid interfaces. The confinement of the lithiation reaction to these specific surface regions is attributed to a reaction‐induced, diffusion‐limiting process (Figure 8e). As shown in Figure 8f, an isotropic compressive strain from 5% to 10% increased the Li^+^ diffusion barrier from 0.4 to 0.8 eV, resulting in a significant 10^7^‐fold reduction in Li^+^ diffusivity in all diffusion directions at the reaction front. Both coin cell and pouch cell (Figure 8g) with the Li/SiG anode, where SiG is the composite layer formed by µSi and graphite particles, a high mass loading LiNi_0.83_Mn_0.06_Co_0.11_O_2_, and a Li_6_PS_5_Cl_1.0_−Li_10_GeP_2_S_12_−Li_6_PS_5_Cl_1.0_ multilayer SE, demonstrated good cycling stability and capacity retention at 6C and 5C and 55 °C, respectively.

Within fast‐charging SSBs, key challenges on the anode side involve maintaining Li metal's structural integrity and preventing undesired mechanical intrusion of Li. Recent studies suggest that elevating stack pressures and temperatures can enhance the maintenance of continuous contact regions, enabling higher charge/discharge rates.^[^ ^19^, ^20^, ^110^ ^]^ Additionally, optimizing the mechanical properties of Li metal, along with leveraging its flow characteristics to alleviate void formation during stripping/plating processes, contributes to improving CCD and rate performance.^[^ ^111^ ^]^ Elements such as Si, Sn, Ge, and Sb can trigger the formation of Li‐containing alloys that offer substantially higher theoretical capacities than conventional carbon materials, showing potential for enhanced energy storage capabilities.^[^ ^112^ ^]^ However, alloy anodes face challenges related to mechanical stability arising from notable volume changes during cycling. Current strategies to address these issues include materials optimization, innovative structural designs, and surface coating developments, aiming to enhance electrode/electrolyte contact, reduce interfacial resistance, and enable more uniform Li plating/stripping, ultimately improving rate capability and cycling stability in SSBs.^[^ ^113^ ^]^

### 4.2 Composite Cathode Materials

Composite cathodes are generally composed of cathode active material (CAM), SE particles, polymer binders, and carbon‐based additives. This integrated assembly increases electrode/electrolyte interfacial conformity, thus enhancing interfacial contact and boosting effective conductivity. Adding highly conductive SE to the cathode helps mitigate tortuosity and residual void issues, particularly in thick electrodes.^[^ ^114^ ^]^ However, large SE particles can inadvertently increase ion transport tortuosity by introducing substantial porosity.^[^ ^115^ ^]^ Consequently, optimizing the cathode‐to‐SE particle size ratio (*λ*) becomes crucial for balancing ion and electron transport. While a large *λ* value enables higher CAM mass loading and enhanced energy density in SSBs, there are practical limitations. Excessively large CAM particles require longer lithiate and delithiate times, while extremely small SE particles increase grain boundaries and reduce percolation channel diameters, leading to higher impedance.^[^ ^115^ ^]^ Therefore, designing optimal composite cathodes for fast‐charging SSBs requires careful consideration of this fundamental trade‐off between power and energy density.

Most CAMs include transition‐metal cations, which mediate charge gain and loss during Li^+^ insertion and extraction. However, low electronic and ionic conductivity can lead to insufficient rate performance. For instance, LiFePO_4_, a widely commercialized cathode, has poor rate capability because of its low electronic conductivity (<10^−9^ S cm^−1^) and Li^+^ diffusion coefficient (10^−14^ to 10^−16^ cm^2^ s^−1^).^[^ ^116^ ^]^ Various strategies, such as conductive nanoscaled coating and nanostructuring, can enhance the rate performance.^[^ ^117^ ^]^ By creating a lithium phosphate coating as a fast ion‐conducting surface phase through controlled off‐stoichiometry on nanoscale LiFePO_4_, Kang and Ceder achieved remarkable capacities of over 100 mAh g^−1^ at 60C and 60 mAh g^−1^ at 400C.^[^ ^10b^ ^]^ At 100C, carbon‐coated single‐crystal LiMn_2_O_4_ nanoparticle clusters demonstrated an impressive capacity of ≈80 mAh g^−1^.^[^ ^118^ ^]^ Compared to LiFePO_4_, the layered oxides, such as LiCoO_2_ and Ni‐rich layered oxides, often realize faster Li^+^ diffusion via 2D channels. An impressive high‐rate capability was realized by constructing LiCoO_2_ with a controlled particle size of 17 nm, reaching 65% of its 1C rate capability at 100C.^[^ ^119^ ^]^ However, these modifications, which are common in conventional LIBs, are severely limited in fast‐charging SSBs. Unlike liquid electrolytes that can penetrate into cathodes, the physical contact between SEs and cathodes is crucial in determining the electrochemical properties of SSBs. Furthermore, even small volumetric changes in CAMs can cause contact loss between cathodes and SEs during charge–discharge cycles, leading to the formation of isolated particles and significant degradation of electrochemical properties. In this regard, exploring potentially promising CAMs and improving ion percolation within the cathode by optimizing cathode microstructure will be critical to achieving fast‐charging SSBs.

**Figure 9:**
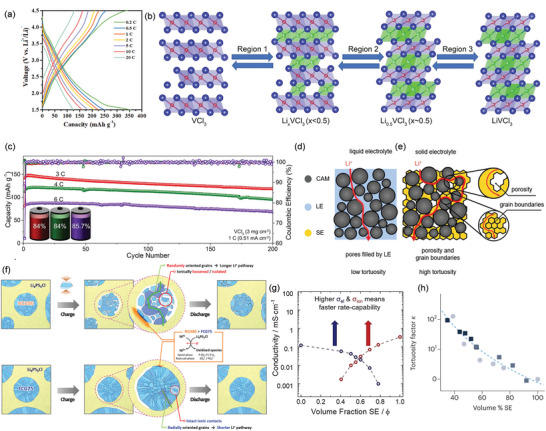
a) Typical charge–discharge curves of a Li/VOPO_4_ solid‐state Li metal battery measured from 0.2C to 20C. Reproduced with permission.^[^ ^121^ ^]^ Copyright 2023, Wiley‐VCH. b) Illustration of the structure evolution of VCl_3_ during lithiation/delthiation. c) Cycling stability of SSBs utilizing a VCl_3_‐Li_3_InCl_6_‐C cathode at 3C, 4C, and 6C. Panels b,c) Reproduced with permission.^[^ ^122^ ^]^ Copyright 2023, Wiley‐VCH. Comparison of composite cathodes using d) liquid‐ or e) solid‐electrolytes. Reproduced with permission.^[^ ^24^ ^]^ Copyright 2022, Wiley‐VCH. f) Schematic illustrating the different microstructural and interfacial evolutions in conventional Li[Ni_0.80_Co_0.16_Al_0.04_]O_2_ (NCA80) CAM, and radially oriented CAM (FCG75) in SSBs. Reproduced with permission.^[^ ^123^ ^]^ Copyright 2019, Wiley‐VCH. g) Schematic improvements of transport with the addition of SE. Reproduced with permission.^[^ ^124^ ^]^ Copyright 2023, Wiley‐VCH. h) Evaluated tortuosity factor as a function of the volume fraction of SE. Reproduced with permission.^[^ ^125^ ^]^ Copyright 2023, Springer.

Efforts are underway to explore alternative CAMs enabling fast‐charging SSBs. For instance, high‐power multielectron pseudocapacitive cathode materials that possess fast ion and electron transport are promising candidates. Transition metal oxide materials, such as V_2_O_5_, Nb_2_O_5_, and TiO_2_ demonstrate pseudocapacitance, a phenomenon where reversible redox reactions take place at or near a material's surface interfacing with an electrolyte, or when these reactions are not restricted by solid‐state ion diffusion.^[^ ^120^ ^]^ Pseudocapacitive materials exhibit rapid charging and discharging behaviors that typically occur within seconds to minutes, thus realizing high energy and power density concurrently. A recent work involved the synthesis of 2D VOPO_4_ nanosheets with enriched V^4+^ defects (VOPO_4_@G‐Air) as a high‐capacity pseudocapacitive cathode.^[^ ^121^ ^]^ Through annealing the precursors in air instead of in O_2_, enriched V^4+^ defects were obtained, offering positive effects for accelerating the Li^+^ diffusion process of VOPO_4_ and reducing polarization. Furthermore, the 2D nanosheet structure provides more open active sites for multielectron reactions, greatly accelerating the multistep Li^+^ intercalation process and improving the kinetics of multielectron reactions. Consequently, the SSB with ultrathin Li anode, as‐synthesized VOPO_4_@G‐Air cathode, and an SE based on ethoxylated trimethylolpropane triacrylate had almost no capacity decay after 250 cycles at 5C, delivering superior rate performance up to 20C (**Figure** **9**a).

Metal halides (such as MX*_n_*, M: Fe, Cu, Ni, etc., and X: F, Cl, and Br) exhibit promising characteristics as battery electrodes, the theoretical capacity of which can reach several hundred mAh g^−1^.^[^ ^126^ ^]^ Although their solubility in liquid electrolytes hinders their use in conventional LIBs, there may be an opportunity to pair metal halide electrodes with SEs. This approach allows for the exploitation of their advantageous properties while overcoming their solubility limitations. The Sun group investigated layered cathodes VX_3_ (X = Cl, Br, I), which are chemically compatible with halide SEs (e.g., Li_3_InCl_6_).^[^ ^122^ ^]^ VX_3_ exhibited a hexagonal‐closed‐packed (hcp) framework with V^3+^ occupying the octahedral holes, wherein the edge‐shared VX_6_ octahedra were stacked in an AB sequence (O1‐type structure, R‐3 space group) along the c direction (Figure 9b, left). The fully lithiated state of LiVCl_3_, characterized by an O3 layered structure with a R‐3m space group (Figure 9b, right), revealed the intercalation of Li^+^ in the Van der Waals interlayers. Thanks to the fast Li^+^ insertion/extraction in the layered VX_3_ and favorable interface guaranteed by the compatible electrode/electrolyte design, the designed SSB, comprising Li_3_InCl_6_ as the SE, VCl_3_‐Li_3_InCl_6_‐C as the cathode, Li metal as the anode, and a protective Li_6_PS_5_Cl layer, exhibited promising performance with long‐term cycling stability and 84%–85.7% capacity retention at 3, 4, and 6C over 200 cycles (Figure 9c).

### 4.3 Design of Electrode Microstructures

Liquid electrolytes can easily transport through the interfaces and penetrate into porous electrodes (Figure 9d), while solid–solid interfaces in SSBs may suffer from poor contact and limited interfacial area, even under high external pressure. This can lead to high interfacial resistance and slow Li^+^ transport kinetics across the interfaces.^[^ ^24^ ^]^ As shown in Figure 9e, the microstructures employing SEs consist of porosity and grain boundaries, which can result in ambiguous and uneven transport properties at the microscale. The high interfacial resistance, slow Li^+^ transport, and long transport distances may cause heterogeneous electrochemical reactions, reduce the utilization of CAMs, and deteriorate the electrochemical performance of SSBs.^[^ ^24^ ^]^ Reducing ion‐path tortuosity is an effective way to accelerate Li^+^ diffusion in thick electrodes.^[^ ^127^ ^]^ This is because the specific capacity and rate capability closely relate to the characteristic diffusion time, τ, given by $\tau =L_{\mathrm{ion}}^{2}/D\;$, where *D* is the diffusion coefficient and *L*_ion_ is the diffusion length. Thus, cathode microstructures are critical for fast‐charging SSBs. For example, in contrast to CAMs featuring randomly oriented grains, CAMs with radially oriented rod‐shaped grains can accommodate volume changes, thereby maintaining mechanical integrity (Figure 9f).^[^ ^123^ ^]^

Optimal Li^+^ transport should follow a direct path from the interior to the exterior of CAMs. However, in conventional secondary particles, randomly oriented primary particles disrupt this ideal migration pathway. This limitation underscores the need to comprehensively explore ionic transport pathways. Secondary particles with oriented grains facilitate radial Li^+^ diffusion along the optimal grain plane, effectively reducing diffusion tortuosity within CAMs.^[^ ^51^, ^128^ ^]^ For instance, Yang et al. developed a vertically‐aligned LiFePO_4_ cathode using an ice‐template freeze‐casting method, which greatly reduced the Li^+^ transport distance by dividing the thick electrode into vertically‐aligned “thin electrodes”.^[^ ^129^ ^]^ This approach resulted in a Li‐LiFePO_4_ battery with a glass fiber‐reinforced composite polymer electrolyte and a vertically‐aligned high‐mass‐loading LiFePO_4_ cathode (10.5 mg cm^−2^), achieving a high areal capacity of 1.52 mAh cm^−2^. Similarly, Grant and co‐authors fabricated a 600 µm‐thick cathode made of vertically aligned NMC811‐rich materials filled with a polymer electrolyte using a directio freezing and polymerization technology.^[^ ^130^ ^]^ This design improved Li^+^ diffusion throughout the cathode from 4.4 × 10^−9^ to 1.4 × 10^−7^ cm^2^ s^−1^. For the interior of CAMs, the implementation of single‐crystal materials offers continuous Li^+^ conduction channels within individual particles. The absence of grain boundaries, potentially enables faster Li^+^ transport.^[^ ^131^ ^]^ The Sun group compared single‐crystal LiNi_0.5_Mn_0.3_Co_0.2_O_2_ (SC‐NMC532) and polycrystalline counterparts using sulfide‐based SSBs, demonstrating higher capacity and better rate performance than those with polycrystalline NMC532 (PC‐NMC532). Han et al. investigated the electrochemical performance of single‐ and poly‐crystalline LiNi_0.88_Co_0.11_Al_0.01_O_2_ cathode‐based SSBs.^[^ ^51^ ^]^ They highlighted the remarkable synergy achieved by combining cracking‐free single‐crystalline LiNi_0.88_Co_0.11_Al_0.01_O_2_ and oxidation‐tolerable Li_3_YCl_6_ electrolyte while emphasizing the importance of often overlooked intercoupled engineering factors, such as particle size, lightness, and mixing. The developed SSBs with single‐crystalline LiNi_0.88_Co_0.11_Al_0.01_O_2_ attained exceptional battery performance with long cycling stability of 200 cycles and high rate capacity of 130 mAh g^−1^ at 4C and 30 °C.

In achieving fast‐charging cathodes for SSBs, the key lies in optimizing ion and electron transport within the composite cathode. Critical considerations include enhancing ionic and electronic conductivity, managing contact losses among the phase boundaries of SE, CAM, and carbon, and understanding complex interphase interactions within the composite cathode during cycling. As shown in Figure 9g, while the higher SE content can increase the ionic conductivity, it simultaneously reduces the electronic conductivity.^[^ ^124^ ^]^ Moreover, the increase of SE content reduces the tortuosity factor, facilitating Li^+^ transport (Figure 9h).^[^ ^125^ ^]^ Therefore, a balanced SE and CAM content in the composite electrode, as well as considering tortuosity are needed to achieve high rate capacities. Strategies such as exploring novel multielectron pseudocapacitive and promoting the formation of single crystals can enhance both the ionic and electronic conductivity of CAM. Additionally, the application of external pressure helps to minimize contact loss and microstructural cracking, ensuring stable cycling performance during fast charging.

## 5 Interfacial Challenges

Despite significant advancements in SEs and electrode materials, fast‐charging SSBs continue to face challenges due to interfacial instabilities between the electrode and electrolyte, especially under high C‐rates. At the anode, the formation of interfacial voids induced by Li stripping determines the morphological instabilities during Li plating and stripping. This is exemplified by dendrite growth that exceeds CCD and serves as a major impediment to achieving high charging rates. On the cathode side, the electrode/electrolyte interface may encounter challenges such as compromised ion transport, reduced electrochemical reactions, and structural degradation under fast‐charging conditions, all of which hamper the overall electrochemical performance of SSBs. Success in developing fast‐charging SSBs depends on the engineering of interfaces that ensure compatibility, along with the stability and efficiency of the SEI and CEI interphases. These interphases must possess high ionic conductivity, be thin and compactly integrated with the electrodes, and maintain electrochemical stability throughout cycling. Therefore, addressing these interfacial challenges is paramount to achieving higher current densities and improved fast‐charging performance in SSBs.

### 5.1 Anode/Electrolyte Interface/Interphase

Interfacial void formation during Li plating and stripping increases battery overpotential and accelerates Li dendrite growth. The microscopic evolution of the Li void can be interpreted by nucleation and growth theory.^[^ ^132^ ^]^ For void nucleation, the nucleation barrier (Δ*G*_V_) and the critical radius of the voids nucleus (*r*_crit_) are represented by^[^ ^133^ ^]^

(1) $$\Delta G_{V}=\frac{-FiR_{s}}{V_{m}}\;$$

(2) $$r_{\mathrm{crit}}=\left|\frac{2\gamma_{\mathrm{Li}}}{\Delta G_{V}}\right|\propto \frac{1}{i}$$

where *F*,  *i*,  *R*_s_,  *V*_m_, and γ_Li_ are the Faraday constant, the current density, the area specific impedance, the molar volume of Li metal, and the specific surface energy of Li metal, respectively. During void growth, linear growth is favored along the interfaces due to positional factors. The void growth rate ($\frac{dr}{dt}$) follows the following expression^[^ ^132^ ^]^

(3) $$\frac{\mathrm{dr}}{\mathrm{dt}}=\alpha v\;\exp\left(-\frac{\frac{-\mathrm{Fi}R_{s}}{V_{m}}+l\cdot \Delta g_{l}}{k_{B}T}\right)$$

with α indicating the amount of the void per area, *v* standing for the vibration frequency, *l* representing the void migration distance, and Δ*g_l_* denoting the migration barrier per unit distance.

**Figure 10:**
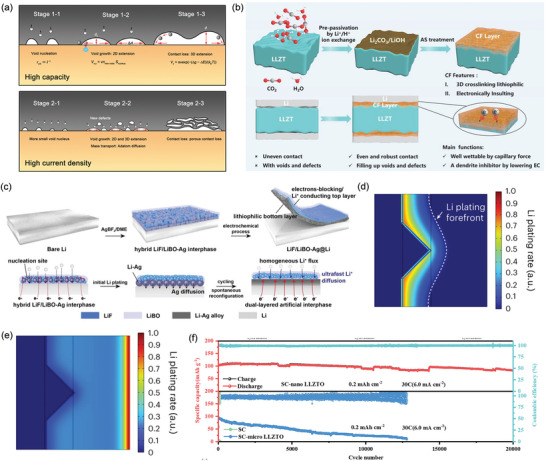
a) Microscopic evolution of voids under high capacity (top) and high current density (bottom) conditions. The process progresses through distinct stages of void nucleation, growth, and contact loss. Reproduced with permission.^[^ ^132^ ^]^ Copyright 2022, The American Association for the Advancement of Science. b) Schematic of the constructing process of Li/CF‐LLZT interface and an illustration of the main functions of CF layer. Reproduced with permission.^[^ ^19b^ ^]^ Copyright 2020, Wiley‐VCH. c) Schematics of the spontaneously reconfigured LiF/LiBO–Ag interphase on a Li foil. Reproduced with permission.^[^ ^134^ ^]^ Copyright 2023, Wiley‐VCH. Simulated Li growth and nucleation in d) mixed conductive and e) electronic conductive interlayers. Reproduced with permission.^[^ ^135^ ^]^ Copyright 2024, Springer. f) Cycling performance of LiZrO_2_‐coated LiCoO_2_|Li_6_PS_5_Cl/SC‐nano/micro LLZTO|Li batteries operated at 30C and 55 °C. Reproduced with permission.^[^ ^136^ ^]^ Copyright 2024, Wiley‐VCH.

**Figure** **10**a illustrates microscopic void evolution.^[^ ^132^ ^]^ At high current density, low *r*_crit_ leads to the formation of diminutive nuclei in abundance, accelerating void growth and contact loss failure. This anodic void accumulation serves as a rate‐determining step for SSBs. The void nucleation process, as outlined in Equations (1) and (2), is governed by interfacial overpotential, which primarily originates from interfacial impedance. Reducing the interfacial impedance, such as improving the initial contact and reducing the interfacial reaction, is efficient in alleviating the subsequent contact loss.

Construction of an artificial SEI using polymers, thin films, or nanoparticles is an effective way to enhance interfacial contact and improve Li^+^ transport in SSBs.^[^ ^137^ ^]^ Interfacial engineering strategies can tune the surfaces of both SEs and anodes. For example, an ultrathin Al_2_O_3_ layer (5 nm) coated on ICE by atomic‐layer deposition (ALD) improved interfacial contact and reduced the interfacial resistance from 1710 to 1 Ω cm^2^.^[^ ^137b^ ^]^ Chen et al. deposited a conformal nanoscale amorphous Al_2_O_3_ coating onto LLZTO by ALD, lowering the sintering temperature of LLZTO and achieving a CCD value of 0.52 mA cm^−2^.^[^ ^138^ ^]^ The formed Li‐Al‐O second phase was electrically insulating but ionically conductive, thus benefiting the fast‐charging performance. Ruan et al. constructed a 3D cross‐linking LiF‐LiCl (CF) layer by acid‐salt treatment on LLZTO.^[^ ^19b^ ^]^ As shown in Figure 10b, the developed lithiophilic and electronic insulating layer improved interfacial contact, suppressed Li dendrite, and reduced interfacial impedance, resulting in a high CCD of 1.8 mA cm^−2^ at 25 °C.

Anode coatings can also reduce interfacial resistance and suppress Li dendrite growth. Huang et al. designed a Li‐C_3_N_4_ composite anode by introducing g‐C_3_N_4_ into the Li metal, resulting in a low interfacial resistance of 11 Ω cm^2^ and a high CCD of 1.5 mA cm^−2^.^[^ ^139^ ^]^ Guo et al. presented a dual‐layer artificial interphase LiF/LiBO‐Ag through the surface reaction between Li and AgBF_4_ (Figure 10c).^[^ ^134^ ^]^ This bilayer interphase, consisting of a heterogeneous LiF/LiBO glassy top layer with ultrafast Li^+^ conductivity and a lithiophilic Li‐Ag alloy bottom layer, synergistically regulated dendrite‐free Li deposition even at an ultrahigh current density of 20 mA cm^−2^. As a result, the quasi‐SSB with the coated Li metal anode and NMC811 cathode delivered a high capacity of 130.7 mAh g^−1^ at 5C, significantly higher than that of the bare Li/NMC811 cell (71.6 mAh g^−1^).

Although electronic conductive and lithiophilic interphases, such as Al and Sn, can suppress void formation by promoting uniform Li deposition, they also accelerate electrolyte reduction due to their high electronic conductivity.^[^ ^137^, ^140^ ^]^ Conversely, lithiophobic and highly ionically conductive interlayers, such as LiF^[^ ^141^ ^]^ and LiF‐Li_3_N,^[^ ^142^ ^]^ can suppress electrolyte reduction by preventing direct contact between Li and the electrolyte, but their limited Li diffusivity may promote interfacial void formation by hindering Li^+^ transport and causing uneven Li deposition. To investigate the relationship among lithiophobicity, electronic/ionic conduction properties of interlayers, and Li dendrite suppression capabilities, the Wang group used the Li_7_N_2_I–carbon nanotube (LNI–CNT) interlayer.^[^ ^135^ ^]^ LNI exhibited high ionic conductivity, low electronic conductivity, and lithiophobic characteristics, while CNT had high lithiophobicity and electronic conductivity but low tap density. Within the mixed conductive LNI–5% CNT interlayer, Li nucleation results in a flat plating forefront for Li plating (Figure 10d), a stark comparison to the uncontrolled Li nucleation observed across the entire electronic conductive interlayer (Figure 10e). As a result, a high CCD of >4.0 mA cm^−2^ was realized in the Li|LNI‐5% CNT|Li symmetric cell. Similarly, the Wu group inserted a hybrid ionic‐electronic conducting layer consisting of soft carbon and nanosized LLZTO between the Li_6_PS_5_Cl SE and the Li anode, and achieved a high CCD of 20 mA cm^−2^ at 0.25 mAh cm^−2^.^[^ ^136^ ^]^ An impressive charge/discharge rate of 175C was realized for the LiZrO_2_‐coated LiCoO_2_|Li_6_PS_5_Cl/SC‐nano/micro LLZTO|Li SSB at 55 °C with a capacity of 40 mAh g^−1^. The full cell exhibited a capacity retention of 80% after 20 000 cycles at a rate of 30C (Figure 10f).

### 5.2 Electrolyte/Cathode Interface/Interphase

Improving the contact area and decreasing the charge‐transfer resistance at the electrolyte/cathode interface/interphase is crucial for realizing fast‐charging SSBs. Several strategies have been employed to enhance the interfacial contact between the cathode and SE, such as designing compatible electrolytes, modifying the cathode structure, and developing composite electrodes, as previously discussed. Alternatively, designing cathode‐supported batteries and introducing a thin Li^+^ conducting oxide buffer layer have proven effective in maximizing the contact area, significantly reducing interfacial resistance, and enhancing interfacial stability.^[^ ^143^ ^]^ These strategies have led to improved rate performance and capacity retention.

**Figure 11:**
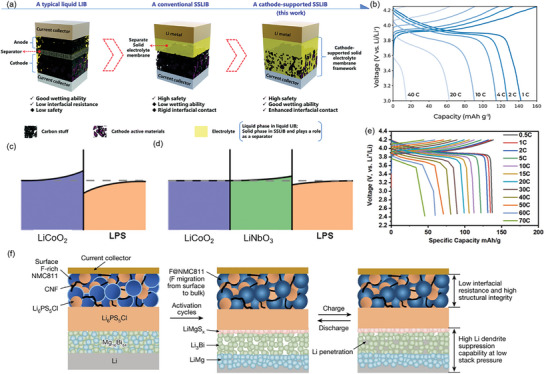
a) Schematics of the cathode‐supported SSB compared to a conventional rigid SSB and a typical LIB. Reproduced with permission.^[^ ^143c^ ^]^ Copyright 2019, The Royal Society of Chemistry. b) Charge–discharge curves at different C‐rates of the LiCoO_2_/Li–Nb–O/LLZO half‐cell with interfacial modification. Reproduced with permission.^[^ ^144^ ^]^ Copyright 2020, American Chemical Society. Li concentration profiles at the LiCoO_2_‐Li_3_PS_4_ interface c) without and d) with an LiNbO_3_ buffer layer. Reproduced with permission.^[^ ^25^ ^]^ Copyright 2014, American Chemical Society. e) Charge–discharge curves of the Li_3_InCl_6_‐coated LiCoO_2_|Li_6_PS_5_Cl|Li battery at different C‐rates. Reproduced with permission.^[^ ^145^ ^]^ Copyright 2024, Elsevier. f) Schematics of the in situ formation of F@NMC811|Li_6_PS_5_Cl/LiMgS*_x_*/Li_3_Bi|LiMg. During the Li plating‐stripping activation cycles, the Mg_16_Bi_84_ interlayer undergoes a transformation into a multifunctional LiMgS*_x_*‐Li_3_Bi‐LiMg triple interlayer, facilitating Li plating on the LiMg surface and penetration into the porous Li_3_Bi. Simultaneously, the surface fluorine‐rich NMC811 cathode convert into F‐doped NMC811 (F@NMC811) due to the electrochemical migration of fluorine anions from the surface to the bulk of NMC811. Reproduced with permission.^[^ ^146^ ^]^ Copyright 2023, Springer.

Chen et al. fabricated a cathode‐supported SE for SSBs by directly casting the PEO‐based SE on the cathode layer to enhance the interfacial contact (**Figure** **11**a).^[^ ^143c^ ^]^ Under this method, the pores inside the cathode layer are filled by the SE, leading to reinforced interfacial adhesion due to capillary attraction. Results demonstrated improved rate capacities compared to conventional SSBs. Sastre et al. investigated the interface between the LLZO SE and the LiCoO_2_ cathode in thin‐film SSBs.^[^ ^144^ ^]^ By coating a 300 nm‐thick amorphous Nb_2_O_5_ layer on the LiCoO_2_ cathode with ALD, the charge transfer resistance between LLZO and LiCoO_2_ was reduced to 50 Ω cm^2^, achieving a threefold reduction compared to previously reported values. The combination of low interfacial resistance and high conductance through the thin‐film LLZO electrolyte allowed high charge–discharge rates up to 40C (Figure 11b). Impressively, the thin‐film SSBs demonstrated discharge capacities of ≈140 mAh g^−1^ at 1C, while 60% of the theoretical capacity was retained for over 100 cycles at 10C.

The strategy of adopting a buffer layer is effective and more commonly used between sulfide SE and cathode due to the low Li^+^ chemical potential and weak attraction compared with a high voltage cathode. When a sulfide SE interacts with LiCoO_2_, Li^+^ migrates from the electrolyte to the cathode, leading to the formation of a high‐resistance Li‐deficient layer at the interface (Figure 11c).^[^ ^25^ ^]^ This region of charge separation detrimentally impacts battery efficiency, limiting their fast charging.^[^ ^147^ ^]^ A thin oxide buffer layer can mitigate direct exposure of sulfides and cathode, and suppress the effect of space‐charge layer (Figure 11d).^[^ ^25^, ^148^ ^]^ Similarly, the Wu group proposed Li_3_InCl_6_‐coated LiCoO_2_ via a freeze‐drying technique.^[^ ^145^ ^]^ The resulting SSBs, configured as Li_3_InCl_6_ coated LiCoO_2_|Li_6_PS_5_Cl|Li, exhibited exceptional electrochemical performance, achieving a capacity of 45 mAh g^−1^ at a charge–discharge rate of 70C (Figure 11e). These SSBs demonstrated stable cycling for 7000 cycles at 20C (9.4 mA cm^−2^) and maintained stable cycling for 100 cycles even at a surface‐specific capacity of 5 mAh cm^−2^. The Wang group proposed an interface design on both cathode and anode sides, with an Mg_16_Bi_84_ interlayer at the Li/Li_6_PS_5_Cl interface to suppress Li dendrite growth, and an F‐rich interlayer on the NMC811 cathode to reduce interfacial resistance.^[^ ^146^ ^]^ The Mg_16_Bi_84_ interlayer underwent conversion during the initial annealing and Li plating‐stripping activation cycles, resulting in a multifunctional LiMgS*_x_*‐Li_3_Bi‐LiMg triple interlayer, and the F‐rich interlayer‐coated NMC811 was converted into F‐doped NMC811 (F@NMC811) (Figure 11f). The anode and cathode interlayer designs enabled the NMC811|Li_6_PS_5_Cl|Li cell to achieve a capacity of 7.2 mAh cm^−2^ at 2.55 mA cm^−2^ at a low stack pressure, and the F@NMC811|Li_6_PS_5_Cl–Mg_16_Bi_84_|Li cell with a CAM loading of 0.51 mAh cm^−2^ delivered a capacity of 86.1 mAh g^−1^ over 681 cycles at 60 °C and 5C.

The electrode/SE interface in SSBs is crucial for fast charging but poses challenges. Resolving these challenges mainly involves enhancing interface contact and improving interface stability. In this section, various strategies are exemplified as ways to improve these two aspects and ion diffusion capabilities. However, due to the complex thermodynamic and kinetic factors at the interface, there is still a lack of understanding of the causes, components, and reaction mechanisms of the interface. Comprehensive approaches, such as utilizing nanoscale interface characterizations to observe interfaces,^[^ ^88^ ^]^ employing computational models to investigate reaction mechanisms,^[^ ^38a^ ^]^ and controlling the crystallographic orientation to understand the mechanism underpinning interface instabilities,^[^ ^149^ ^]^ enable the rational design and optimization of electrode/SE interfaces, advancing the development of high‐performance, fast‐charging SSBs.

## 6 Fast Charging Facilitated by Computational Simulations

Computations, encompassing DFT, MD, HTS, and continuum models, offer insights into the thermodynamic, kinetic, and interfacial properties governing fast‐charging SSBs. These techniques, augmented by ML's data‐driven capabilities, expedite material discovery, guide electrolyte design, and assess electrochemical stability and interfacial phenomena, thereby accelerating the development of high‐performance fast‐charging SSBs.

### 6.1 Ion Transport Mechanism

**Figure 12:**
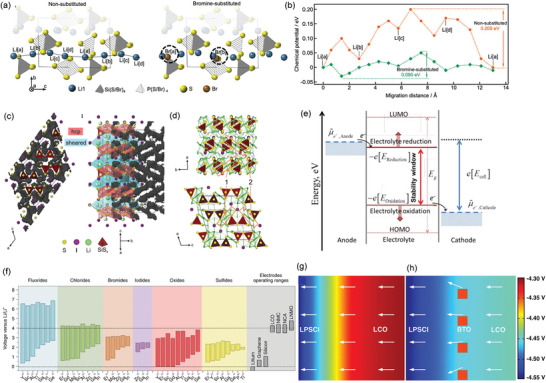
a) Calculated migration energy barriers for the models without and with bromine substitution. b) Potential energy profile along a Li^+^ migration pathway in LSiGePSBrO. Panels a,b) Reproduced with permission.^[^ ^152^ ^]^ Copyright 2024, The American Association for the Advancement of Science. c) Projections of the Li_7_Si_2_S_7_I supercell along the [010] and [101] directions, and d) migration pathways. Reproduced with permission.^[^ ^153^ ^]^ Copyright 2024, The American Association for the Advancement of Science. e) Negative and positive potential limits for electrolyte stability, along with the energy levels of HOMO and LUMO. Reproduced with permission.^[^ ^157^ ^]^ Copyright 2018, The Royal Society of Chemistry. f) HOMO and LUMO energies for commonly used SEs and electrodes. Reproduced with permission.^[^ ^158^ ^]^ Copyright 2020, Springer. g,h) Simulation results of the internal electrical field for the LiCoO_2_/Li_6_PS_5_Cl and BaTiO_3_–LiCoO_2_/Li_6_PS_5_Cl interfaces. The arrows represent the direction of the internal electrical field. Reproduced with permission.^[^ ^159^ ^]^ Copyright 2020, Springer.

Researchers often employ the nudged elastic band (NEB) or the climbing image NEB (CI‐NEB) methods to simulate diffusion. NEB calculations serve to determine the energy barrier and transitional state when tracking the migration pathway of a mobile ion. This migration typically takes place in the presence of a vacancy or interstitial, facilitating the transition of the ion from one stable site to another. The application of NEB techniques has notably enhanced the comprehension of the static energy landscape related to ion migration pathways in both electrodes and SE materials.^[^ ^35^, ^150^ ^]^ However, these calculations must be conducted at the dilute limits for single vacancies or hops, necessitating prior knowledge of the diffusion channels. This becomes much more complicated for many ICEs especially with highly disordered Li^+^ sublattices. Ab initio MD (AIMD) simulations, conducted to observe migration events directly, can offer input for NEB calculations and provide intricate, atomistic‐level information regarding ion diffusion.^[^ ^151^ ^]^ For instance, Li et al. proposed a novel concept for developing high‐entropy SEs with exceptional Li^+^ conductivity while preserving the desired crystal structure.^[^ ^152^ ^]^ By calculating the energy barrier of ion migration (**Figure** **12**a,b), they demonstrated that minor chemical substitutions in Li_10_GeP_2_S_12_‐type Li_9.54_Si_1.74_P_1.44_S_11.7_Cl_0.3_ (i.e., Li_9.54_[Si_0.6_Ge_0.4_]_1.74_P_1.44_S_11.1_Br_0.3_O_0.6_ (LSiGePSBrO)) effectively reduced the Li^+^ migration barrier. Consequently, the synthetic monophasic LSiGePSBrO achieved a high ionic conductivity of 32 mS cm^−1^ at 25 °C and the corresponding symmetric Li cell with a Li|Li_10.25_P_3_S_12.25_I_0.75_‐LSiGePSBrO‐Li_10.25_P_3_S_12.25_I_0.75_|Li configuration exhibited a CCD of 3.3 mA cm^−2^ at 60 °C. Han et al. successfully synthesized a novel SE known as Li_7_Si_2_S_7_I with a high RT ionic conductivity of 10 mS cm^−1^.^[^ ^153^ ^]^ AIMD simulations were employed to study the Li^+^ transport in Li_7_Si_2_S_7_I, the corresponding network and migration pathways are shown in Figure 12c,d. As shown in Figure 12c, there are 162 distinct site‐to‐site connections, with absolute energy barriers below 0.3 eV. Among these connections, 11 of them (including two types of units) have energy barriers even lower than 0.2 eV.

By analyzing MD simulations, we can gain insights into transport mechanisms and design SEs with enhanced conductivity. The self‐diffusivity (*D*_Li_), a key parameter in characterizing the diffusion of mobile ions in SEs, can be calculated as follows^[^ ^154^ ^]^

(4) $$D_{\mathrm{Li}}=\lim_{t\to \infty }\frac{1}{2\mathrm{dNt}}\sum_{i=1}^{N}{\left|r_{i}\left(t\right)-r_{i}\left(0\right)\right|}^{2}\;\;$$

where *d* is equal to 3 for typical crystals. *r_i_* (0) and *r_i_* (*t*) represent the initial positions of the *i*‐th mobile ion (out of *N*) and position at time *t*, respectively. *N* is the number of diffusing atoms. Using the self‐diffusion coefficient, the ionic conductivity can be calculated as follows^[^ ^69^ ^]^

(5) $$\sigma =D\left(Ne^{2}/Vk_{B}T\right)$$

where *k*_B_ is the Boltzmann constant, *V* is the system volume, and *T* is the temperature. For example, the Li^+^ ionic conductivity of ≈10–14 mS cm^−1^ in Li_10_GeP_2_S_12_, as determined through AIMD simulations, aligns closely with the experimental measure of 12 mS cm^−1^.^[^ ^151^, ^155^ ^]^ Klerk et al. examined MD simulations on β‐Li_3_PS_4_ to demonstrate that jumps between *bc* planes limited the conductivity. The simulations indicated that the rate‐limiting jump process can be accelerated significantly by Li interstitials or Li vacancies; correspondingly, promoting 3D diffusion, resulting in increased macroscopic Li^+^ diffusivity.^[^ ^156^ ^]^ We compared the diffusivity and ionic conductivity of a QSPE and liquid electrolyte using AIMD simulation. The results demonstrated that the developed QSPE had better diffusion and higher ionic conductivity at low temperature, which is in agreement with the experimental values.^[^ ^69^ ^]^

### 6.2 (Electro) Chemical Interfacial Stability

Interfacial impedance between the SE and electrode remains a significant challenge for fast‐charging SSBs. Fast charging induces mechanical stress and chemical reactions at these interfaces, forming detrimental SEI layers that impede ion transport. The Goodenough group proposed evaluating electrolyte stability relative to electrodes by calculating the energy difference between the Fermi level of the electrode and the highest occupied molecular orbital (HOMO) or lowest unoccupied molecular orbital (LUMO) levels of the electrolyte.^[^ ^160^ ^]^ The HOMO level of the electrolyte should be lower than the Fermi level of the cathode and the LUMO level should be higher than the Fermi level of the anode to prevent unwanted reactions (Figure 12e).^[^ ^157^ ^]^ Figure 12f depicts the electrochemical stability windows of selected SEs in comparison to common electrode materials.^[^ ^158^ ^]^ DFT calculations are widely used to assess LUMO–HOMO energies and electrolyte/electrode compatibility. Li et al. utilized DFT to design a polymer electrolyte leading to stable, conductive dual‐layered SEI and uniform CEI, resulting in SSBs with excellent rate performance up to 10C.^[^ ^68^ ^]^ Similarly, Liu et al. applied DFT to analyze the LUMO–HOMO energy of the electrolyte components between the electrode and the electrolyte composition, revealing the formation mechanism of inorganic compound‐intensive CEI and LiF‐rich gradient SEI layers.^[^ ^161^ ^]^ The optimized electrolyte demonstrated excellent electrochemical compatibility with the high‐voltage NMC811 cathode, effectively suppressing Li dendrite formation and enabling high‐rate (5C) performance in SSBs.

HTS has emerged for the identification of liquid electrolytes, oxide‐based electrode coatings, and SEs.^[^ ^162^ ^]^ Ren et al. proposed a workflow for screening interface coatings from 17 082 Li‐contained compounds obtained from the Material Project,^[^ ^163^ ^]^ utilizing criteria, such as electronic insulation, phase stability, chemical stability, and ionic conductivity.^[^ ^143a^ ^]^ The approach identified 48 candidate coatings between the oxide cathodes and sulfide SEs, exhibiting favorable interfacial compatibility and high ionic conductivity, thereby improving Li^+^ transport at the interface. In another study, the Ceder group employed HTS to select Li‐containing materials as cathode coatings for SSBs, focusing on their phase stability, electrochemical and chemical stability, and ionic conductivity.^[^ ^162b^ ^]^ Through this screening process, polyanionic oxide coatings, including LiH_2_PO_4_, LiTi_2_(PO_4_)_3_, and LiPO_3_, emerged as particularly promising candidates. Li et al. employed high‐throughput first‐principle calculations in the search for protective‐layer materials among 2316 Li‐containing compounds.^[^ ^164^ ^]^ They identified 5, 28, and 7 materials suitable for protecting LLZO, Li_3_PS_4_, and LiTi_2_(PO_4_)_3_, respectively. The protective layer effectively blocks electron transfer from the Li metal to the SE, thereby mitigating dendrite growth. These studies demonstrate the potential of HTS in discovering interface‐stable coatings for SSBs, which is crucial for enabling fast charging and improving overall battery performance

Despite significant theoretical and experimental progress, a comprehensive understanding of interfacial phenomena in SSBs remains a critical challenge. In particular, elucidating the interplay of ion transport, charge distribution, and interfacial deformations caused by volume changes between the electrode and SE, especially under fast‐charging conditions, is essential for advancing SSBs. Continuum models have emerged as valuable tools for simulating the intricate physical and chemical processes at these interfaces, including ion and electron transfer, interface reactions, diffusion, and electric field distribution. For instance, Wang et al. employed continuum models to investigate the impact of discontinuous BaTiO_3_ nanoparticles coating on a LiCoO_2_ cathode, demonstrating improved interfacial stability and rate performance.^[^ ^159^ ^]^ As shown in Figure 12g, an obvious interface internal electrical field was found from cathode to SE at the LiCoO_2_/Li_6_PS_5_Cl interface, while the gradient was greatly reduced after coating BaTiO_3_ (Figure 12h), suggesting the reduced space‐charge‐layer and improved interfacial stability. The Cui group combined continuum models with experimental characterizations to reveal the detrimental effects of mechanical deformation on ion flux and cycling performance at the Li anode/Li_10_SnP_2_S_12_ interface.^[^ ^165^ ^]^ These examples underscore the importance of understanding interfacial properties in SSBs and the potential of continuum models, alongside experimental techniques, to unravel the complex phenomena occurring at the electrode/electrolyte interface, paving the way for the design of fast‐charging SSBs.

### 6.3 High‐Throughput Screening‐ and Machine Learning‐Assisted Materials Discovery

HTS is aemerging as a valuable tool for expediting the discovery of new materials, particularly SEs. While conventional experimental techniques and first‐principles simulations remain essential, their inherent limitations in rapidly assessing extensive chemical spaces necessitate alternative approaches. HTS provides a streamlined methodology for the evaluation of numerous candidate materials, facilitating the identification of those exhibiting desirable properties.

**Figure 13:**
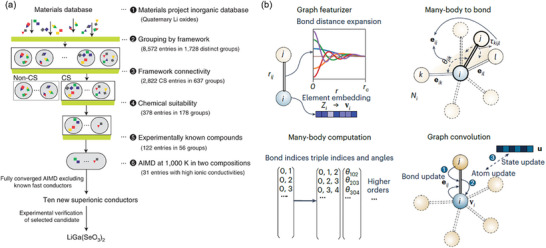
a) Flowchart of the multistep computational screening. Reproduced with permission.^[^ ^33^ ^]^ Copyright 2022, Springer. b) Schematic of the major computational blocks of M3GNet. The graph featurizer illustrates element embedding into a learnable continuous feature space and bond distance expansion into a basis set with values and derivatives of up to second order, going to zero at the boundary. The many‐body computation module, incorporating the many‐body to bond module and the graph convolution. The many‐body to bond computation derives three‐ and many‐body interaction atom indices and the associated angles. Reproduced with permission.^[^ ^167^ ^]^ Copyright 2022, Springer.

A notable example of HTS implementation is the development of a comprehensive database encompassing crystal structure information, ion migration channel connectivity, and 3D channel maps for over 29 000 inorganic compounds.^[^ ^166^ ^]^ This database not only speeds up the screening process for fast ionic conductors, but also provides accumulated descriptors for ML algorithms. Moreover, the efficacy of HTS is exemplified by a multistep screening approach employed to identify potential Li^+^ superionic conductors.^[^ ^33^ ^]^ As shown in **Figure** **13**a, commencing with an expansive dataset of quaternary lithium oxides, materials were systematically classified into distinct structure groups based on their framework geometry. Subsequent rigorous filtering steps, including polyhedra connectivity analysis, bandgap assessment, and elemental suitability considerations, substantially narrowed the search space. The application of AIMD simulations enabled the calculation of conductivity and activation energy for the remaining candidates, ultimately identifying novel oxide frameworks exhibiting superionic conductivity. Notably, one such material (i.e., LiGa(SeO_3_)_2_) has been experimentally validated, demonstrating high ionic conductivity and low activation energy. These exemplary cases highlight the transformative potential of HTS in driving materials discovery for SSBs. By enabling the efficient exploration of vast chemical spaces and leveraging data‐driven methodologies, HTS could significantly accelerate the identification and development of high‐performance materials.

ML has also emerged as a powerful tool for materials discovery, leveraging its ability to recognize complex patterns in data and utilizing critical descriptors, such as composition and crystal structure to identify materials with desired properties. Chen et al. proposed M3GNet, a graph neural network‐based interatomic potential (IAP), to accurately describe the potential energy surface of atoms and enable atomistic simulations.^[^ ^167^ ^]^ M3GNet enriches the representation of atomic environments by capturing n‐body interactions through distinct combinations of neighboring atoms, enabling the modeling of high‐order interactions, such as angles and dihedrals (Figure 13b). Trained on extensive databases of materials properties and corresponding relaxed structures, this model is capable of predicting material stability and performing MD simulations with DFT accuracy. Deng et al. proposed CHGNet, another graph neural networked‐based IAP, which models the universal potential energy surface.^[^ ^168^ ^]^ This model enables fast structure optimization and provides site‐wise magnetic moments, making it ideal for pre‐relaxation and initialization of magnetic moments in spin‐polarized DFT calculations, as well as charge‐informed MD. These models can be further fine‐tuned or used for new IAP training, accelerating the discovery of novel materials with exceptional properties. To address the issue of labeled data scarcity, Zhang et al. proposed unsupervised learning techniques as a valuable tool in materials discovery.^[^ ^169^ ^]^ By leveraging limited conductivity data, unsupervised learning algorithms prioritize candidates for further screening. This approach has led to the identification of 16 new fast Li‐conductors with the AIMD‐predicted Li^+^ conductivity ranging from 10^−1^ to 1 mS cm^−1^. These materials exhibit unique structures and chemistries, highlighting the capability of unsupervised learning to explore a wide range of compounds with limited property data.

ML has also been applied to automate precursor selection in solid‐state synthesis. Algorithms such as ARROWS^3^ actively learn from experimental outcomes to identify optimal precursor combinations that avoid the formation of unwanted intermediates.^[^ ^170^ ^]^ By minimizing the formation of stable intermediates, ARROWS^3^ proposes new experiments that retain a larger thermodynamic driving force, facilitating the synthesis of target materials.^[^ ^170^ ^]^ This approach has proven effective in reducing the number of experimental iterations while identifying effective precursor sets. Integrating ARROW^3^ with HTS algorithms and ML models can significantly accelerate the discovery of electrode materials with high capacity and stability, as well as SEs with high ionic conductivity.

### 6.4 Modeling in Rate Performance

As early as 2010, Notten and colleagues introduced a 1D mathematical model for SSBs.^[^ ^171^ ^]^ Their simulations, reaching a rate as high as 51.2C, highlighted that overpotential primarily stems from transport constraints within the SE. In 2023, Shen et al. established a 2D model of SSBs to study their rate performance under low temperatures.^[^ ^172^ ^]^ Li^+^ migration in SE predominantly limits discharge rates. By reducing the thickness of SE and enhancing Li^+^ transport can boost high‐current discharge performance in SSBs. Furthermore, Fathiannasab et al. designed a 3D model for SSBs.^[^ ^173^ ^]^ By incorporating electrode microstructure into the 3D model, increased ohmic losses were anticipated on the electrodes. Particularly at elevated current densities, the electrode's microstructure significantly influenced the electrochemical properties, indicating that low ionic and electronic conductivity, along with low tortuosity, are pivotal factors for enhancing fast‐charging capabilities in SSBs.

## 7 Advanced Characterization Technology

The widespread adoption of SSBs is currently limited by two key challenges: the poor ionic conductivity of SEs and the incompatibility between electrodes and SEs. To unravel the intricate (electro)chemical/physical processes, researchers have extensively employed advanced characterization techniques, including synchrotron X‐ray techniques, neutron diffraction (ND), and solid‐state nuclear magnetic resonance (SS NMR).^[^ ^174^ ^]^ This review explores how these advanced characterizations have enhanced our understanding of SEs and their interfaces, aiming to stimulate further research and advance the development of fast‐charging SSBs.

### 7.1 Synchrotron X‐Ray

High‐resolution synchrotron characterization enables detailed examination of various phenomena in SEs, including phase evolution and crystallization mechanisms,^[^ ^175^ ^]^ stress evolution,^[^ ^26^ ^]^ and atomic‐to‐nanoscale features in thin films and interfaces,^[^ ^176^ ^]^ paving the way for tailored designs that optimize both electrochemical performance and stability in SSBs. For instance, the utilization of operando X‐ray tomography has allowed researchers to observe void formation and interphase growth in Li/Li_10_SnP_2_S_12_/Li symmetric cells at high current densities (≥ 1 mA cm^−2^), revealing that current constrictions caused by interfacial void formation and contact loss are crucial factors contributing to battery failure.^[^ ^177^ ^]^ Pan et al. paid attention to the interface contact between electrodes/IPCs, Li^+^ migration, and electrochemical reactions, and proposed the development of multiscale characterization technologies using large scientific devices (such as synchrotron radiation), in situ visualization confocal microscopy, and in situ secondary ion mass spectrometry to detect interface changes.^[^ ^178^ ^]^

### 7.2 Neutron Diffraction

Similar to X‐ray diffraction, ND measures the atomic structure of materials but offers greater sensitivity to light elements such as Li and H. Therefore, neutron‐based techniques are important and powerful structural and analytical tools for SSBs. Wu et al. employed an operando ND measure to investigate structural evolution and transition dynamics of electrodes under different C rates, identifying the rate‐limiting step during fast‐charging.^[^ ^179^ ^]^ ND also aids in determining the structural models of SEs, encompassing crystals,^[^ ^180^ ^]^ crystalline‐amorphous composites,^[^ ^181^ ^]^ and amorphous structures.^[^ ^43^ ^]^ These insights not only elucidate the structural complexity of SEs, but also provide a solid foundation for understanding their high ionic conductivity. ND has enabled direct visualization of Li spatial distribution in a solid‐state Li–S battery, revealing that sluggish macroscopic ion transport within the composite cathode is the rate‐limiting factor.^[^ ^182^ ^]^

### 7.3 Solid‐State NMR

SS NMR excels in investigating atomic‐scale carrier transport characteristics and has gained considerable attention for exploring SEs and ion movement at interfaces/interphases. For instance, Liu et al. investigated Li^+^ transport pathways within IPCs using electrochemical impedance spectroscopy and NMR techniques, discovering that Li^+^ transport at ceramic/polymer interphase depends on the interface structure.^[^ ^91^ ^]^ Zheng and co‐workers combined isotope exchange with NMR to map the Li^+^ diffusion routes in IPCs, demonstrating that Li^+^ predominantly transports pathways through the LLZO phase.^[^ ^183^ ^]^ Our previous work investigated the potential Li^+^ transportof IPCs by SS NMR, demonstrating multiple Li^+^ diffusion pathways.^[^ ^57c^ ^]^ SS NMR provides valuable insights into how local interface/interphase environments between inorganic and organic components in SEs influence ion transport, offering opportunities to develop materials with high ionic conductivity essential for fast‐charging SSBs.

SSBs are in the early developmental stages, and the ionic conductivity of many optimized SEs can meet the requirements for SSB applications.^[^ ^184^ ^]^ Consequently, SSBs research focus has shifted from enhancing the ionic conductivity of SEs to tackling interface issues. The cycling process involves diverse components and complex evolutionary patterns, making advanced characterization techniques essential for understanding the relationship between structure and electrochemical performance. Nevertheless, each advanced technique has its limitations. Combining the aforementioned characterization methods with other technologies—including in situ Raman, in situ microscopy, operando stress measurement, and cryogenic electron microscopy—enables more comprehensive understanding of interface/interphase chemistry and ion movement.

## 8 Industry Breakthroughs

SSBs have emerged as the preferred candidate for next generation battery technology due to their enhanced safety, high energy density, superior power characteristics, and broad temperature adaptability. While mass production of SSBs remains unrealized, several companies have successfully demonstrated prototypes (**Table** **4**). There is a noticeable shift in SSB development focus from energy density and cycle life improvements to fast‐charging capabilities, aiming to enhance consumer experience through reduced charging times. This section outlines the current progress and strategies of global battery companies in fast‐charging SSB development, with the emphasis on accelerating industrialization.

**Table 4:** Details of fast‐charging SSBs disclosed by some companies.

Company	Electrolyte	Cathode	Anode	Operating temperature	Energy density	Capacity	Charge rate	Source
Mache Power	Sulfide	High‐nickel cathode	C	60 °C	210 Wh kg^−1^	≈1 Ah	10C	Social media
Enpower Greentech	Sulfide	NMC	Li	−40–100 °C	300 Wh kg^−1^	1–10 Ah	5C	Social media
Ampcera	Sulfide	NMC	Si	RT	400 Wh kg^−1^	/	4C	Company website
Solid Power	Sulfide	NMC	Si	70 °C	470 Wh kg^−1^	20 Ah	5C	Social media
Samsung SDI	Sulfide	NMC	Anode‐free	60 °C	900 Wh L^−1^	/	5C	Company website
Prologium	Oxide+Liquid Electrolyte (<10%)	Li‐free soft cathode	Ultrathin Li metal anode	/	500 Wh kg^−1^	/	5C	Company website
Qingtao Energy Development	Oxide+Polymer+Liquid Electrolyte (=10%)	High‐nickel cathode	Si‐C	/	360 Wh kg^−1^	180–240 Ah	/	Social media
Farasis Energy	Oxide	LiFePO_4_	/	/	200–240 Wh kg^−1^	/	4C	Social media
QuantumScape	Oxide	NMC	Anode‐free Li metal	25–45 °C	/	/	4C	Company website

### 8.1 Sulfide‐Based Technology Route

Multiple companies are pioneering fast‐charging SSB development using sulfide‐based technology routes. Enpower Greentech Inc. developed 1–10 Ah sulfide‐based SSB prototypes in the middle of 2023, realizing a high energy density of 300 Wh kg^−1^. Their latest semi‐SSB operates stably between −40 and 100 °C, supports charging/discharging above 5C, and exhibits a 20% capacity decay after 1000 cycles at RT and 1C.^[^ ^185^ ^]^ Ampcera Inc. has unveiled an SSB technology featuring sulfide‐based SEs, high‐capacity NMC cathodes, and silicon‐based anodes, achieving an impressive energy density of 400 Wh kg^−1^.^[^ ^186^ ^]^ This SSB design, free of liquid or semisolid components, prioritizes safety during fast charging. The technology achieves 0%–80% SOC in 15 min, maintaining less than 5% capacity decay after 300 cycles. Solid Power Inc. has developed sulfide‐based SSBs with a similar battery configuration, recharging 90% of their capacity in 10 min.^[^ ^187^ ^]^ Japanese and Korean companies also investigate the sulfide technology route. Toyota, researching SSBs for decades, recently discovered new materials to realize technology breakthroughs. While details remain limited, they claim its breakthrough on batteries will hit the market in 2027 or 2028, giving its EVs 746 miles of range with 10‐min charging times.^[^ ^188^ ^]^ Samsung SDI's “Dream Battery”, offering an energy density of 900 Wh L^−1^ and 80% charge capability in just 9 min, plannes to large‐scale production by 2027.^[^ ^189^ ^]^

### 8.2 Oxide‐Based Technology Route

The oxide‐based technology route has also attracted the attention of many companies for SSB industrialization. Due to the rigidity of ceramic materials, current developments primarily focus on semisolid designs. Prologium has pioneered batteries featuring an ultrathin Li metal anode, ICE, and Li‐free soft cathode, achieving an impressive gravimetric energy density of 500 Wh kg^−1^. With less than 10% liquid electrolyte, this battery delivers rapid charging, reaching from 5% to 80% in 9 min and 5% to 60% in 5 min.^[^ ^190^ ^]^ WeLion New Energy adopted oxide‐based SEs with in situ polymerization technology, launching a fast‐charging SSB prototype with 270 Wh kg^−1^ for drones in 2019. This prototype features 5C charging/discharging capabilities and entered mass production in 2020.^[^ ^191^ ^]^ Qingtao Energy Development Group Co., Ltd provides semi‐SSBs with the configuration of silicon‐carbon anode, high‐nickel cathode, and composite SE for EVs, achieving a peak charging power of 400 kW.^[^ ^192^ ^]^ Farasis Energy announced a super pouch solution in 2024, featuring a semi‐SSB prototype. When paired with an LFP cathode, this system provides a range of 400 km in 10 min. The prototype SSB from QuantumScape's laboratory maintains over 80% capacity after 400 cycles of 4C charging and 1C discharging, achieving a rapid 10%–80% charge in less than 15 min.^[^ ^193^ ^]^ StoreDot has unveiled its “100inX” strategic roadmap for extreme fast‐charging battery technology.^[^ ^194^ ^]^ Their development timeline includes 100 miles in 5 min by 2024, 100 miles in 3 min by 2028, and 100 miles in 2 min by 2032.

Global interest and investment in fast‐charging SSB development continues to accelerate. Spanning major automotive manufacturers to innovative startups, this expanding ecosystem drives technological advancement in the field. The ongoing research momentum sets a robust stage for future breakthroughs and the commercialization of SSB technology, accelerating the path toward widespread EV applications.

## 9 Summary and Perspectives

This review examines the scientific challenges of ion and electron transport within SSBs while highlighting recent advances in material design, interface engineering, and electrolyte optimization for fast‐charging applications. We also discuss developments in computational methodologies, advanced characterization technologies, and industry breakthroughs in fast‐charging SSBs. Although significant progress has been made in this field, numerous challenges and obstacles remain for practical implementation. We put forward some future research directions and prospects, aiming to accelerate the development of fast‐charging SSBs.

1. The ionic conductivity of SEs stands as the cornerstone of their functionality, playing a pivotal role in enhancing the fast‐charging performance of SSBs. Employing a combination of theoretical calculations and experimental methods is instrumental in identifying novel Li superionic conductors or optimizing the conductivity of existing SEs. While thinner SEs reduce internal resistance, thereby improving the rate performance and power density of SSBs, achieving thicknesses comparable to commercial LIBs′ polymer separators remains challenging. Although reducing SE thickness can lower SSB costs and improve commercial viability, it places greater demands on mechanical properties, as thinner SEs must withstand fractures and potential Li dendrite infiltration during fast cycling. Future research should focus on innovative material design and engineering strategies that balance minimal thickness with mechanical robustness, ensuring both fast ion transport and the stable and long‐term performance of SSBs.
2. The electrochemical window requirements for SEs fundamentally differ from traditional liquid electrolytes. While liquid electrolytes must maintain stability across both electrodes due to their permeability, the inherent rigidity of SEs enables a more targeted design, focusing stability considerations primarily on either anode or cathode electrode interface. This advantage has led to the development of asymmetric or multilayer SE architectures that independently stabilize either anode or cathode.^[^ ^195^ ^]^ These asymmetric structures satisfy specific stability requirements at each electrode, eliminating the need for additional buffer layers or interface coatings. A notable demonstration of this approach employed a multilayer SE combining Li_5.5_PS_4.5_Cl_1.5_ and Li_9.54_Si_1.74_(P_0.9_Sb_0.1_)_1.44_S_11.7_Cl_0.3_ with a graphite‐covered Li anode (Li/G) and NMC811 cathode. This Li/G|Li_9.54_Si_1.74(_P_0.9_Sb_0.1_)_1.44_S_11.7_Cl_0.3_‐Li_5.5_PS_4.5_Cl_1.5_‐Li_9.54_Si_1.74_(P_0.9_Sb_0.1_)_1.44_S_11.7_Cl_0.3_|NMC811 SSB exhibited exceptional rate performance between 0.5C and 20C, maintaining 82% capacity retention after 10 000 cycles at a 20C rate and 55 °C.^[^ ^16b^ ^]^ The development of such highly conductive inorganic asymmetric SEs represents a promising avenue for fast‐charging SSBs, warranting further research into design and optimization strategies.
3. High‐theoretical‐capacity Li anode enables reduced electrode thickness and shorter charge transport distances. However, challenges, such as Li dendrite growth, void formation, and decomposition reactions at the anode/SE interface hinder the achievement of high current densities. Alternative alloy‐type anodes, such as Si, show promise but introduce new challenges related to uncontrolled morphological changes. Various strategies, including material optimization and structural design, can help alleviate these challenges and enhance fast‐charging performance.^[^ ^99^, ^101^ ^]^ Defect‐induced Nb_2_O_5_ materials show enhanced fast‐charging characteristics and cycle stability.^[^ ^196^ ^]^ Recently, the Nb_1.60_Ti_0.32_W_0.08_O_5−δ_ anode, engineered by introducing Ti and W into the Nb_2_O_5_ structure, showcased a remarkable discharge/charge current density of 45 mA cm^−2^ under a stacking pressure of 60 MPa at 60 °C, thanks to the in situ formation of a thin film of lithiated WS_2_ at the Li_6_PS_5_Cl/NMC811 interphase.^[^ ^197^ ^]^ The evolution of battery technology indicates that true advancement is not merely about substituting anode materials but it involves developing “anode‐free” batteries, which should be more accurately denoted as “zero excess Li metal” batteries.^[^ ^198^ ^]^ Minimizing the anode lowers internal resistance, enabling faster charging and discharging rates, which is crucial for applications requiring high power output. Moreover, “zero excess Li metal” anode can mitigate safety concerns related to dendrite formation, a common issue in Li batteries that may trigger short circuits. This forward‐looking strategy presents a promising direction for fast‐charging SSBs.
4. Contrary to current trends favoring a high CAMs content (>80 wt%) and thick cathode architectures, fast‐charging SSBs require a high proportion of SE to ensure sufficient and rapid ion transport in the cathode design. Meanwhile, an electrode with a thin cathode architecture is preferred as it shortens the ion and electron transport distance, facilitating fast charging. While the high proportion of SE and thin cathode architecture constrain the batteries’ energy density, creating a fundamental trade‐off between power density and energy density which must be carefully considered in application. Optimizing the microstructure of the cathode, crystal plane orientation of CAM, the size or distribution of the cathode composites, and conductive coatings help overcome the power/energy trade‐off issue in SSBs, making it a compelling direction for future research in fast‐charging SSBs.
5. Although SSBs offer enhanced safety compared to conventional liquid electrolyte‐based LIBs, they are not entirely risk‐free. Contrary to common perception, SEs can be susceptible to thermal runaway, particularly when in contact with highly reactive materials, such as Li metal and high‐nickel cathodes. For instance, some oxide‐based SEs, such as NASICON‐type materials, exhibit violent combustion when making contact with Li.^[^ ^199^ ^]^ The limited strain accommodation in SSBs leads to more significant local mechanical stress and volume expansion compared to LIBs. The presence of uneven ion transport occurring at grain boundaries and interfaces aggravates localized stress within the battery, potentially leading to crack propagation among cell components. Studies on the interfacial thermal stability between sulfides and cathodes reveal that interfacial decomposition is often initiated at temperatures lower than either component's individual threshold, accompanied by the generation of gas/volatiles and heat release.^[^ ^200^ ^]^ Furthermore, internal short‐circuits can be easily triggered by the penetration resulting from the accumulation of the deposited metallic Li in grain boundaries and at the anode surface. These combined factors can rapidly escalate into safety concerns in fast‐charging SSBs. Therefore, investigating the potential hazards linked to thermal runaways by nail penetration, hot box, short‐circuit and so on are essential, and there is an urgent demand for specialized and advanced technologies devoted to analyzing the thermal safety of fast‐charging SSBs.

## Conflict of Interest

The authors declare no conflict of interest.
